# High-Energy-Density Materials: An Amphoteric N-Rich Bis(triazole) and Salts of Its Cationic and Anionic Species

**DOI:** 10.1021/acs.inorgchem.1c02002

**Published:** 2021-10-12

**Authors:** Emmanuele Parisi, Alessandro Landi, Sandra Fusco, Carla Manfredi, Andrea Peluso, Sabrina Wahler, Thomas M. Klapötke, Roberto Centore

**Affiliations:** † Department of Chemical Sciences, University of Naples Federico II, Via Cinthia, I-80126 Naples, Italy; ‡ Department of Chemistry and Biology, University of Salerno, Via Giovanni Paolo II, 132, I-84084 Fisciano, Salerno, Italy; § Department of Chemistry, Energetic Materials Research, Ludwig-Maximilian University, Butenandtstrasse 5−13, D-81377 Münich, Germany

## Abstract

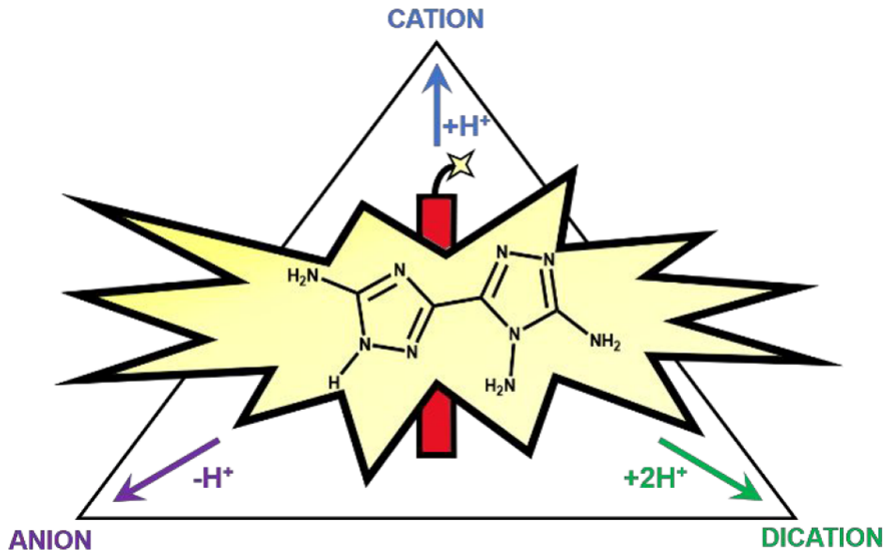

The synthesis and characterization of the N-rich bis(triazole) compound 1*H*,4′*H*-[3,3′-bis(1,2,4-triazole)]-4′,5,5′-triamine (C_4_H_7_N_9_) with a N content of 69.6% by weight is reported. The compound exhibits a rich acid–base behavior because it can accept up to two protons, forming a monocation and a dication, and can lose one proton, forming an anion. Measurement of the acid constants has shown that there exist well-defined pH intervals in which each of the four species is predominant in solution, opening the way to their isolation and characterization by single-crystal X-ray analysis as salts with different counterions. Some energetic salts of the monocation or dication containing oxidizing inorganic counterions (dinitramide, perchlorate, and nitrate) were also prepared and characterized in the solid state for their sensitivity. In particular, the neutral compound shows a very remarkable thermal stability in air, with *T*_d_ = 347 °C, and is insensitive to impact and friction. Salts of the dication with energetic counterions, in particular perchlorate and nitrate, show increased sensitivities and reduced thermal stability. The salt of the monocation with dinitramide as the counterion outperforms other dinitramide salts reported in the literature because of its higher thermal stability (*T*_d_ = 230 °C in air) and friction insensitiveness.

## Abstract 2

An energetic, N-rich compound that, depending on the pH, can exist as neutral, singly protonated, doubly protonated, and deprotonated species is shown.

## Introduction

High-energy-density materials (HEDMs) can store and release in a controllable manner a high amount of (chemical) energy; thus, they are widely exploited in military and civil areas.^1^ When undergoing decomposition, energetic materials produce energy by oxidation processes. One basic problem with HEDMs is that compounds highly performing from the energetic side are often sensitive to external stimuli such as heat, impact, friction, and detonation, requiring some sort of stabilization to control the energy release and avoid accidents. Recently, various classes of heterocyclic compounds with high N content have attracted considerable interest for the development of HEDMs as an alternative to traditional materials because of their excellent stability, high heat of formation, and environmentally friendly conditions.^2^ The average bond energy of the N–N triple bond (954 kJ/mol) is very high, which makes N-rich compounds very endothermic and, therefore, very energetic materials. Also, N-rich heterocycles generally contain N in negative oxidation states, and these materials can decompose, giving environmentally benign gases (i.e., mainly N_2_). Another advantage of N-rich heterocycles is the presence of basic N atoms or acidic N–H groups, which can lead to the formation of coordination compounds or salts in which the N-rich heterocycle is present as a cation or an anion. These salts have intrinsically low volatility and an increased energy content coming from the high energy of the ionic lattice, and their properties can be tuned, in principle, by the appropriate choice of the counterion and by crystal engineering strategies.

**Chart 1:**
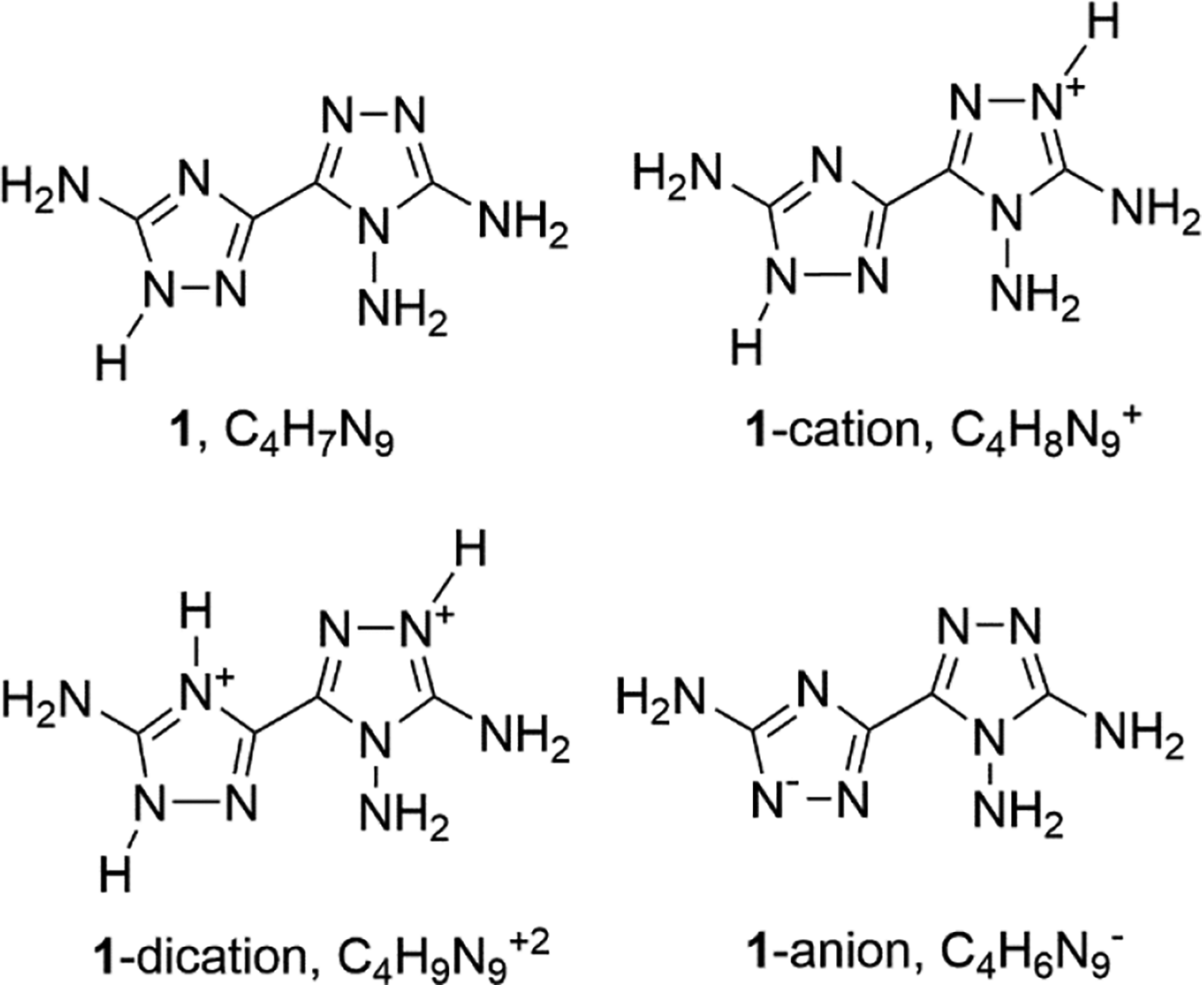
Neutral, Singly Protonated, Doubly Protonated, and Deprotonated Forms of **1**a

Here we report on the synthesis and characterization of HEDMs based on the N-rich heterocyclic compound 1*H*,4′*H*-[3,3′-bis(1,2,4-triazole)]-4′,5,5′-triamine (henceforth compound **1**) shown in Chart 1. It belongs to the class of 1,2,4-triazoles, which have been widely explored in recent years,^3−9^ and has a high N content (69.6%).

a Only one tautomeric form is shown for the neutral and cationic species. For the anion, only one resonance form is shown.

The electron-rich character of **1**, which contains three amino electron-donor groups, is compatible with a rich acid–base behavior. In principle, **1** can take up to two protons, forming a cation and a dication, while the acidic N–H H atom can be lost by reaction with bases, with formation of the anion (Chart 1). So, at variance with most of the energetic N-rich compounds studied so far, with **1**, it is possible, in principle, to prepare salts in which the N-rich heterocycle is present as a cation/dication and salts in which it is present as an anion. Indeed, those salts have been prepared and will be described in the present paper, with full characterization of their properties in the solution and solid state, including measurements of the sensitivities in the solid state for the energetic compounds. Some energetic salts of the **1** dication (nitrate and perchlorate) have recently been studied, independently from us, by the groups of Shreeve^10^ and Cheng/Yang,^11^ with possible application as gas-generating agents, propellants, or explosives. We note that a compound similar to **1** but containing one fewer NH_2_ group (% N 67.4) was used by Shreeve in 2010,^12^ while another one containing one more NH_2_ group (% N 71.4) was described by us.^3,5,6^

## Results and Discussion

### Tautomerism

**Chart 2:**
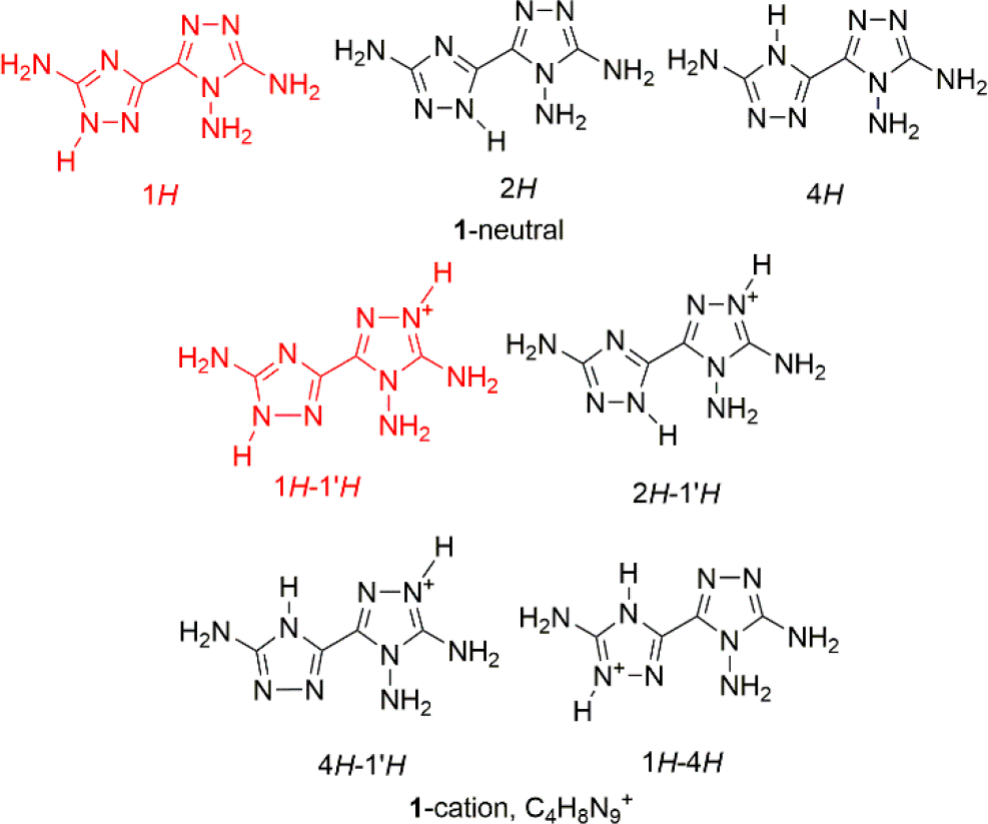
Some Possible Tautomers of **1** and of Its Singly Protonated Cation (Only s-trans Conformers Are Shown)a

Tautomerism is a phenomenon common to several classes of N-containing aromatic heterocycles, exhibiting many intriguing aspects that are relevant in many areas, including crystal engineering,^13^ drug design,^14^ energetic materials,^15^ and coordination chemistry.^16,17^ Of particular interest are compounds for which quasi-degenerate tautomers are possible because they can be switched between each other depending on the environment.^18,19^ The N-rich system of **1** is potentially tautomeric. In Chart 2 are reported the canonical tautomers of the neutral and singly protonated species. Moreover, two different conformers can be expected for each tautomer, differing by the relative orientation of the triazole rings, which can be s-trans or s-cis if we look at the bond between the two rings.

a Highlighted in red are the tautomers/conformers predicted as the most stable by computational analysis.

The computed relative energies of the tautomers/conformers of Chart 2 are shown in Table 1. For the neutral molecule, the most stable predicted species, in a polar medium, is 1*H*/s-trans. The 2*H* tautomer, however, follows quite closely in energy both in the s-trans and, mostly, in the s-cis conformation. In the gas phase, 2*H*/s-cis is predicted as the most stable species, probably as a result of an intramolecular N–H···N interaction, and in a polar medium, the energy of 2*H*/s-cis is only 0.5 kcal/mol higher than 1*H*/s-trans, a value that is within the accuracy of the method used in the calculations. On the other hand, the 4*H* conformer has significantly higher energy.

**Table 1:** Computed Relative Energies (kcal/mol) of Tautomers/Conformers of Neutral and Singly Protonated **1**

	gas		water	
tautomer	s-cis	s-trans	s-cis	s-trans
1*H*	6.7	6.3	0.4	0.0
2*H*	0.0	9.2	0.5	1.3
4*H*	18.2	6.4	5.2	3.5
1*H*–1′*H*	0.4	0.0	0.5	0.0
2*H*–1′*H*	3.6	not stable^a^	3.0	7.1
4*H*–1′*H*	23.2	7.2	7.6	5.0
1*H*–4*H*	18.2	13.8	6.1	7.8

a Interconverts in the conformer s-cis upon geometry optimization.

Concerning singly protonated species, the data of Table 1 indicate that the tautomer 1*H*–1′*H* is the most stable both in the gas phase and in a polar medium. The other tautomers all have significantly higher energy. For the doubly protonated cation, we have not performed any computation. In fact, the tautomer shown in Chart 1 is only possible when both positive charges are on the N atoms adjacent to the C-NH_2_ groups.

### Acid–Base Equilibria in Solution

**Figure 1:**
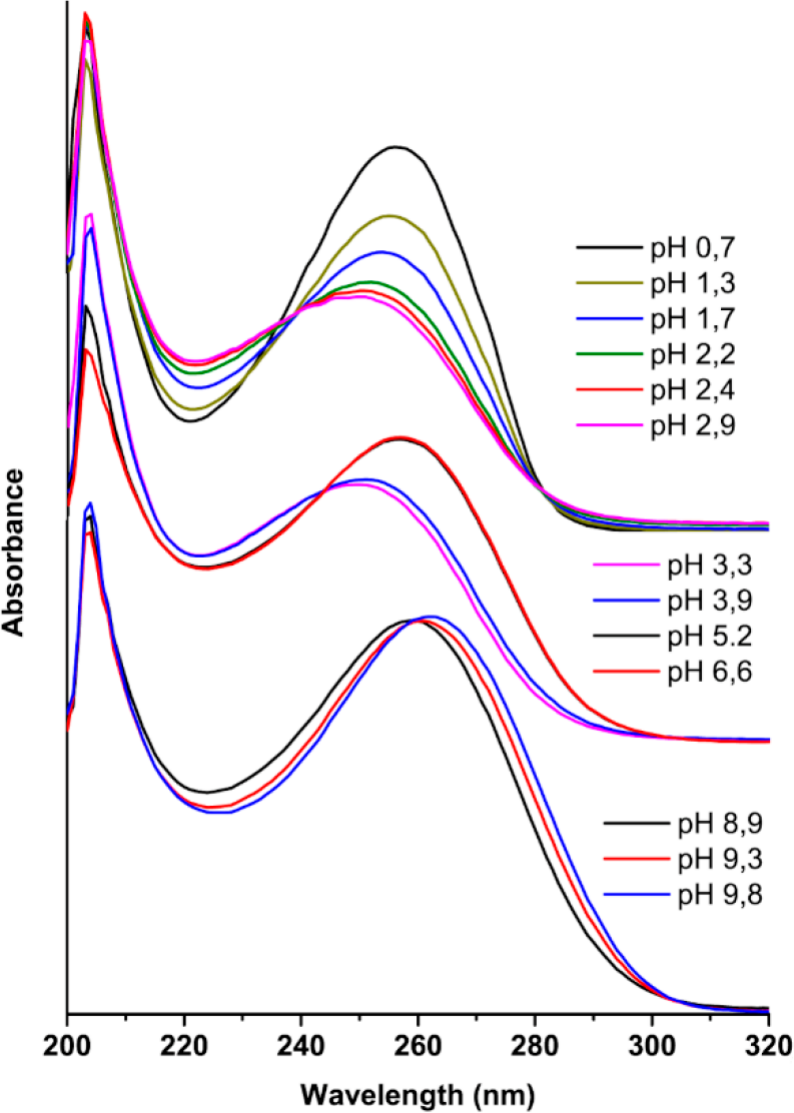
UV–vis absorption spectra of **1** at constant total concentration *c* = 5.01 × 10^–5^ M in 0.5 M NaCl recorded at 0.7 ≤ pH ≤ 9.8. The spectra have been grouped into three sets of curves arbitrarily shifted along the vertical axis for easier lecture. The three sets correspond to the three equilibria involved (*vide ultra* and the SI).

The acid–base equilibria of **1** in solution have been studied at 25 °C by potentiometric–spectrophotometric titrations in the constant ionic medium 0.5 M NaCl (see also the Supporting Information, SI). UV–vis absorption spectra of **1** recorded at different pH values and a constant total concentration are reported in Figure 1.

There is a nonmonotonic dependence of λ_max_ from the pH. Starting from the lowest value of the pH (pH = 0.7, λ_max_ = 257 nm), there is an initial hypsochromic shift up to pH = 2.9 (λ_max_ = 248 nm). Then, with increasing pH, the shift of λ_max_ is always bathochromic up to pH = 6.6 (λ_max_ = 257 nm) and further on to pH = 9.8 (λ_max_ = 264 nm). The changes in the absorption spectra can be accounted for by the three equilibria of Table 2 (see also the SI): neutral **1** (HL) can accept up to two protons, forming the cationic species H_2_L^+^ and H_3_L^2+^, and can release one proton, forming the species L^–^.

**Table 2:** Acid Constants in the Form of p*K*_a_ (at 25 °C in 0.5 M NaCl, with Estimated Standard Deviations in Parentheses) for **1** (HL)

equilibrium	p*K*_a_
H_3_L^2+^ + H_2_O = H_3_O^+^ + H_2_L^+^	p*K*_a__1_ = 1.31(2)
H_2_L^+^ + H_2_O = H_3_O^+^ + HL	p*K*_a__2_ = 4.56(2)
HL + H_2_O = H_3_O^+^ + L^–^	p*K*_a__3_ = 9.25(5)

**Figure 2:**
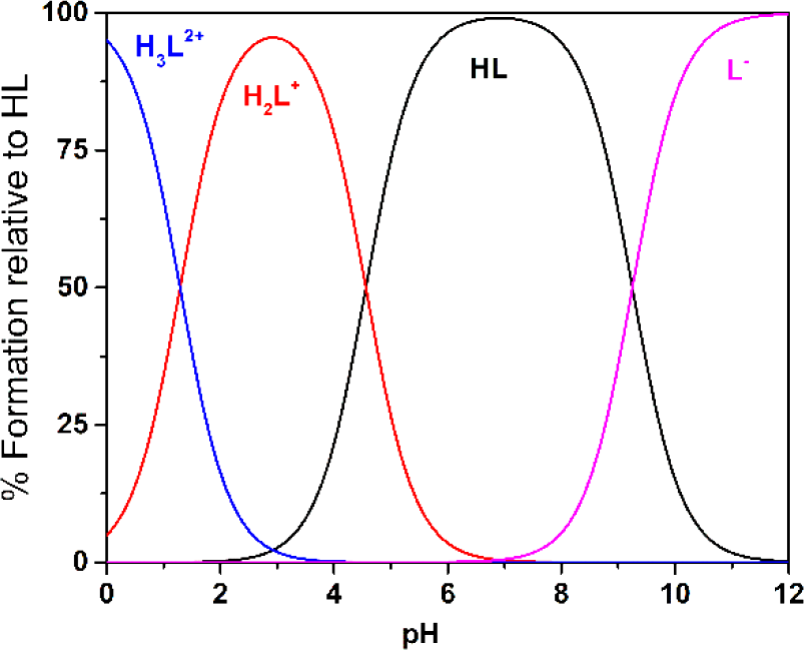
Distribution diagram of **1** (HL), calculated using the constants of Table 2.

The distribution diagram of the species is reported in Figure 2. It is noteworthy that there exist definite pH intervals in which each of the four species involved in the protolytic equilibria is present in solution at a molar fraction of ≥90%. This should allow salts containing each of the three ionic species of **1** to be isolated from solutions.

### Structural and Solid-State Analysis of **1** and Its Salts

**Chart 3:**
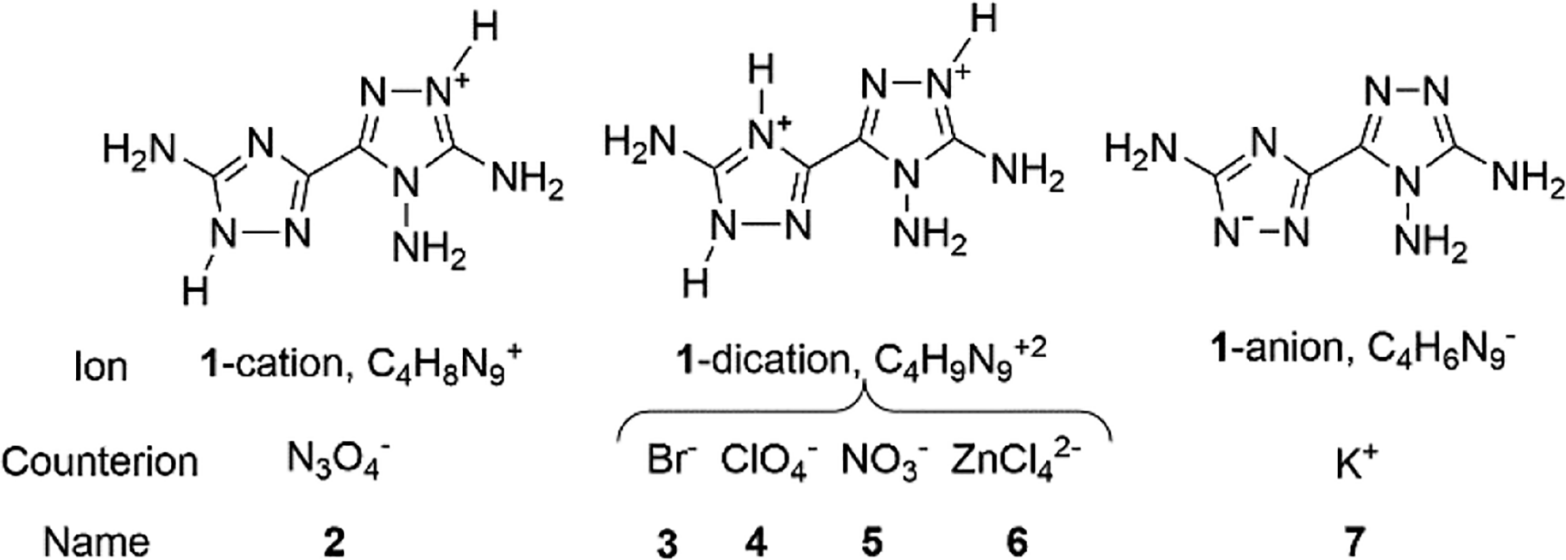
Composition and Numbering of Salts of **1**

We successfully crystallized neutral **1**, **1** monocation as the dinitramide salt, (C_4_H_8_N_9_)(N_3_O_4_), **1** dication as bromide, perchlorate, nitrate, and tetrachlorozincate salts, (C_4_H_9_N_9_)Br_2_, (C_4_H_9_N_9_)(ClO_4_)_2_, (C_4_H_9_N_9_)(NO_3_)_2_, and (C_4_H_9_N_9_)(ZnCl_4_), respectively, and **1** anion as the potassium salt, K(C_4_H_6_N_9_) (Chart 3).

For these compounds, single-crystal X-ray diffraction analysis was performed. Remarkably, the molecular structures of all of the species involved in the acid–base equilibria have been characterized. As a general remark, we note that neutral **1** and its singly and doubly protonated species (Chart 1) are characterized by the presence of several strong H-bonding donor and acceptors on the rim of the molecule. Most of these groups, for instance, the ring N atoms and the C-NH_2_ or N^+^-H groups, are strong in-plane H-bonding acceptors/donors. So, a tendency toward the formation of stacked structures, in which infinite planar layers of H-bonded molecules are piled on each other, is expected for these compounds.

**Figure 3:**
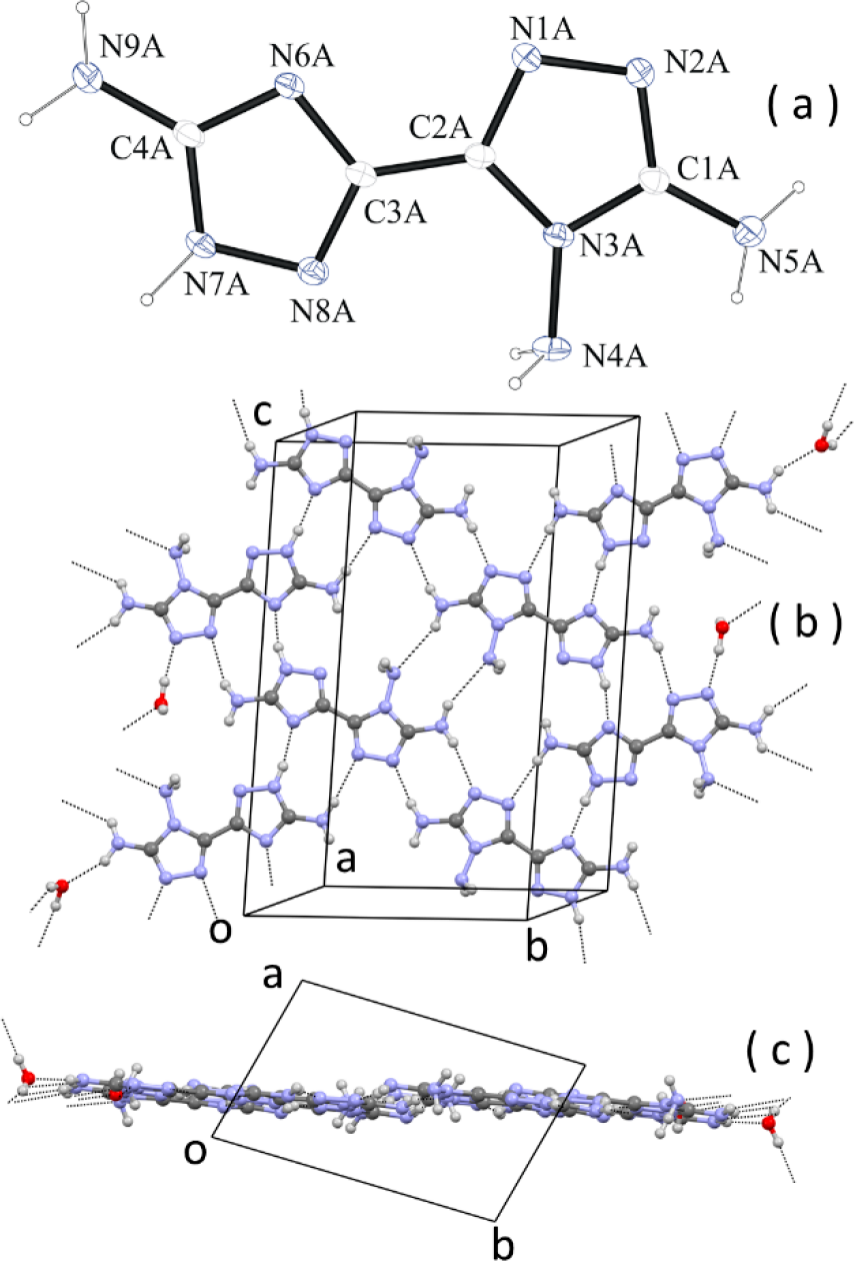
(a) ORTEP diagram of one of the four crystallographically independent molecules of C_4_H_7_N_9_·0.5H_2_O. (b) Face view of a layer of H-bonded molecules. (c) Same layer viewed along *c*. Some H bonds are indicated by dashed lines.

Neutral **1** has been crystallized as a hemihydrate, C_4_H_7_N_9_·0.5H_2_O. Crystals are triclinic *P*1̅ with *Z* = 8. The crystallographic analysis unambiguously indicates that the tautomer present in the crystals is 1*H* (Figure 3a). The four crystallographically independent molecules, all in the s-trans 1*H* tautomer, have a basically flat conformation, with the dihedral angle between the average planes of the two pentatomic rings ranging between 4.2(1)° for molecule A and 11.8(1)° for molecule D. The bond geometry around amino N atoms is relevant for the packing because they are H-bonding donors. The geometry is pyramidal for (N)-NH_2_ atoms [the sum of the valence angles around the amino N atom ranges between 320(3)° and 327(2)° for the four independent molecules]. This result basically remains unchanged in all of the structures studied (*vide ultra*). The geometry around the (C)-NH_2_ N atoms is still pyramidal, but more flat, because of conjugation with the aromatic ring, with the sum of the valence angles around the amino N atom ranging, in this case, between 341(2)° and 358(3)° for the four independent molecules.

Molecules in the crystal form infinite planar layers through H bonds involving the many N–H donor and N acceptors present in the molecules of **1** and the water molecules (Figure 3b,c). The layers are parallel to the lattice plane 21̅0, and, in fact, reflection 21̅0 is the most intense of the whole diffraction pattern. The stacking of the layers is achieved through H bonds between adjacent layers, and the stacking distance of the planes is rather short, *d*_21̅0_ = 3.12 Å. The extended network of strong H bonds accounts for the relatively high density of the crystal, which is 1.629 g/cm^3^ at −100 °C.

**Figure 4:**
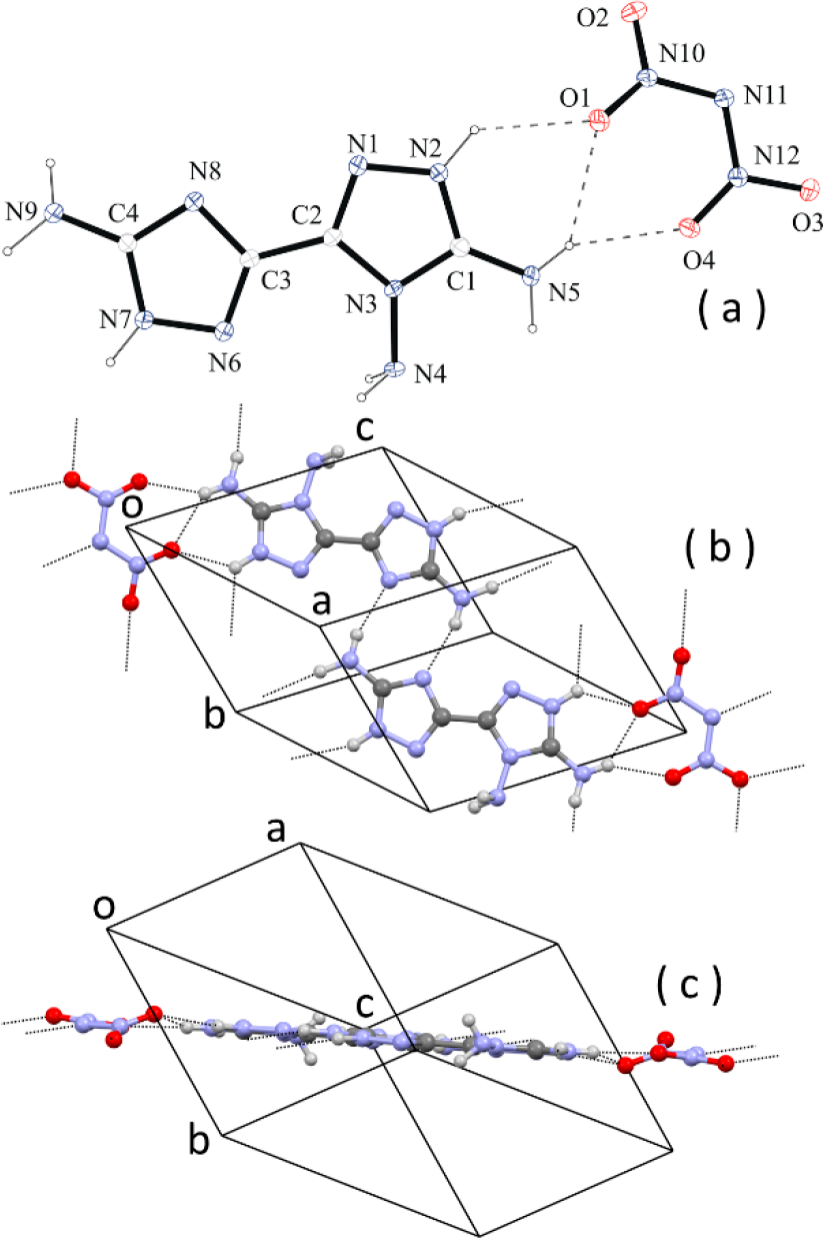
(a) ORTEP diagram of the crystallographic independent unit of (C_4_H_8_N_9_)(N_3_O_4_). (b) Face view of a layer of H-bonded molecules. (c) Edge view of the same layer. Some H bonds are indicated by dashed lines.

By metathesis of the perchlorate salt, (C_4_H_9_N_9_)(ClO_4_)_2_, with potassium dinitramide (KN_3_O_4_), we have obtained the dinitramide salt of monoprotonated **1**, i.e., compound **2** of Chart 3. The crystal structure is shown in Figure 4.

The cation is present as the 1*H*–1′*H* tautomer (Figure 4a), consistent with the results of computational analysis, so single protonation of **1** is observed at the triazole ring bearing two amino groups. The conformation of the cation is basically flat, with the dihedral angle between the average planes of the two rings being 5.2(2)°. The geometry around the amino (N)-NH_2_ atom is pyramidal [the sum of the valence angles is 319(4)°]. Compared with neutral **1**, full planarization of the geometry around the (C)-NH_2_ N atoms is observed, with the sum of the valence angles being 360(5)° and 359(5)°. The packing shows similarities with neutral **1**. Also in this case, molecules form planar ionic/H-bonded layers through N–H donor and N acceptors present in the cation molecule and O and N acceptors of the dinitramide anion (Figure 4b,c). The layers are parallel to the lattice plane 1̅21 (1̅21 is the most intense reflection of the diffraction pattern). The stacking distance of the planes is shorter than **1**, *d*_1̅21_ = 3.11 Å. The extended network of strong H bonds and the ionic interactions between cations and anions account for the significantly higher density of the crystal, which is 1.837 g/cm^3^ at −100 °C.

The salts in which the triamine molecule is present as a dication, i.e., compounds **3**–**6** of Chart 3, were easily prepared by dissolving neutral **1** in a water solution of the corresponding strong inorganic acid (hydrobromic, perchloric, and nitric). In all of the salts studied, the tautomer present is 1*H*–1′*H*–4*H* s-trans (Chart 1). So, protonation (single or double) of neutral **1** is always at the ring N atoms. This is expected because the electron-donor character of the amino groups can stabilize the positive charge of the cation; as a result, the geometry around the amino (C)-NH_2_ N atoms is trigonal-planar in all of the structures of the dications studied.

**Figure 5:**
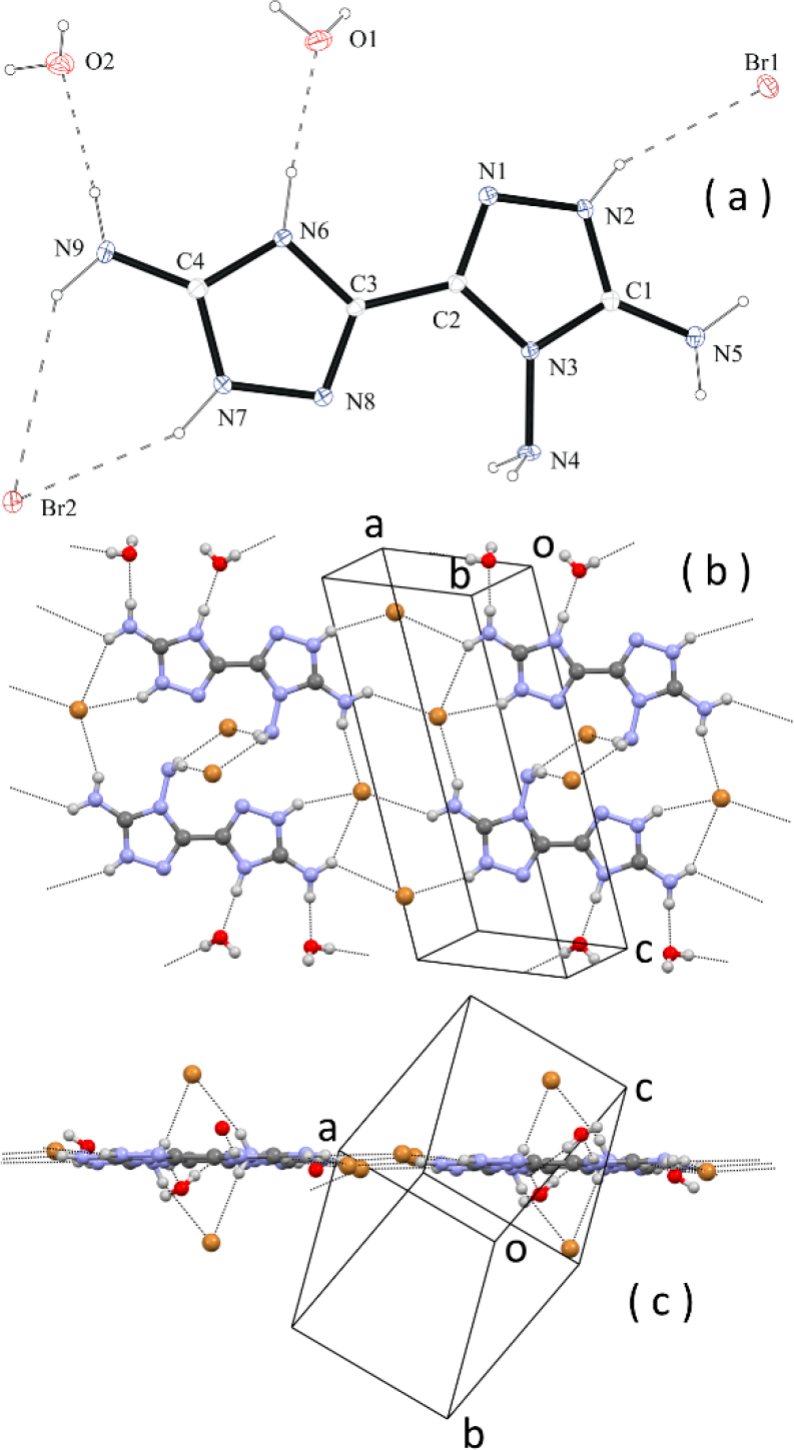
Crystal structure of (C_4_H_9_N_9_)Br_2_·2H_2_O: (a) ORTEP diagram of the crystallographic independent unit; (b) face view of a layer of ionic/H-bonded molecules; (c) edge view of the same layer. Some H bonds are indicated by dashed lines. Some hanging H bonds have been omitted for clarity.

Some features of the crystal structure of the bromide salt are shown in Figure 5.

Again, the structure is of the stacked-layer type. The layers of ionic/H-bonded molecules (Figure 5b,c) are parallel to the lattice plane 12̅2, with a short stacking distance of *d*_12̅2_ = 3.13 Å. The stacking between the layers is accomplished through strong H bonds between bromide ion acceptors and N-NH_2_ donors, with the formation of *R*_4_^2^(8) ring patterns (Figure 5c).

**Figure 6:**
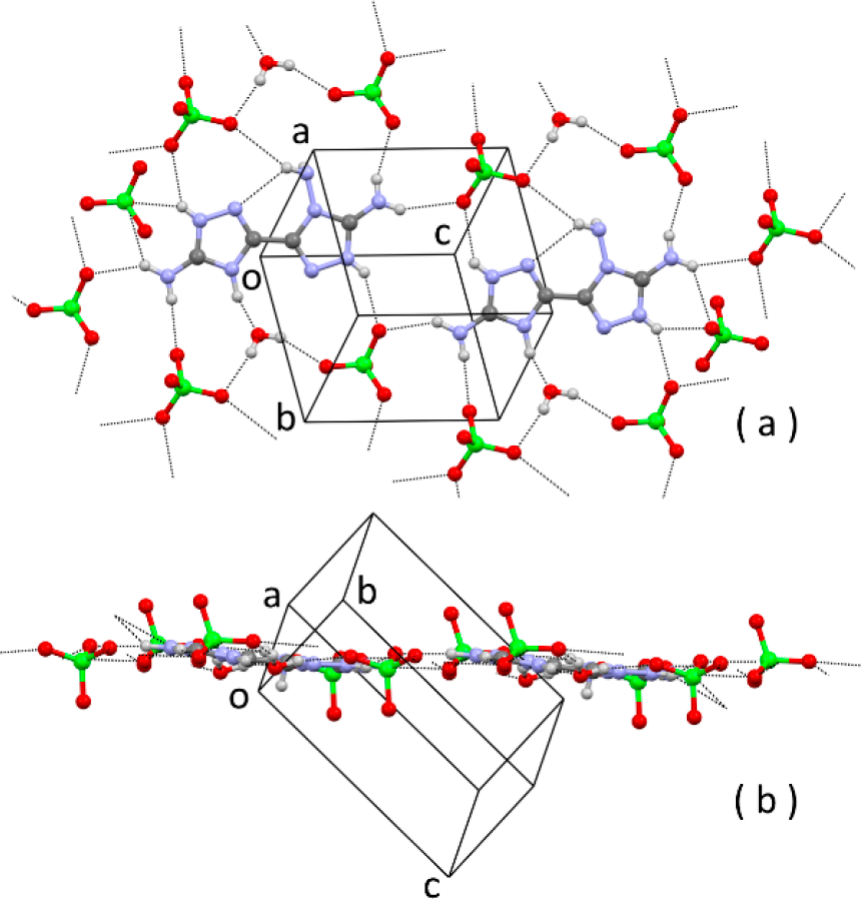
Partial packing of (C_4_H_9_N_9_)(ClO_4_)_2_·H_2_O: (a) face view of a layer of ionic/H-bonded molecules; (b) edge view of the same layer. H bonds are indicated by dashed lines. Some hanging H bonds have been omitted for clarity.

The energetic perchlorate salt is interesting because in the crystal the N-rich dication is fully surrounded by oxidizing tetrahedral perchlorate anions. The conformation of the dication is again flat, with the dihedral angle between the average planes of the two rings being 5.8(2)°. The layers of ionic/H-bonded molecules (Figure 6a,b) are parallel to the lattice plane 112̅, with a stacking distance of *d*_112̅_ = 3.22 Å.

**Figure 7:**
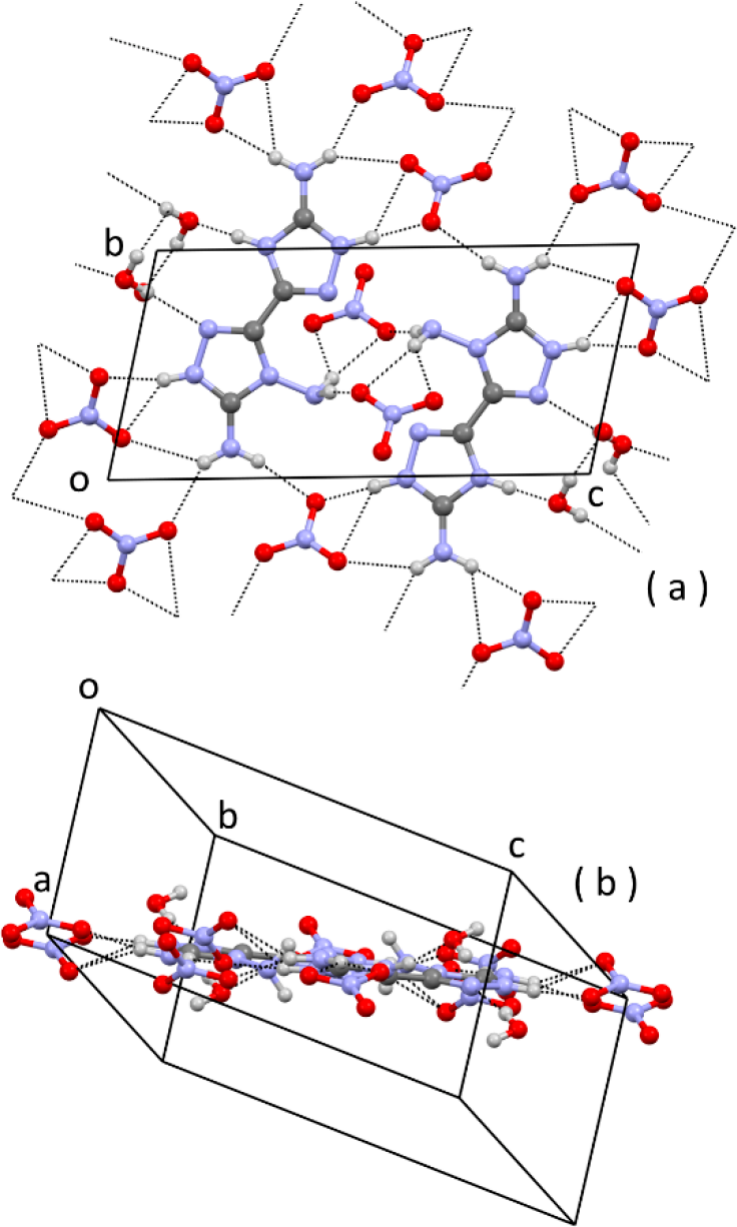
Partial packing of (C_4_H_9_N_9_)(NO_3_)_2_·H_2_O: (a) face view, along *a*, of a layer of ionic/H-bonded molecules; (b) edge view of the same layer. H bonds are indicated by dashed lines. Some hanging H bonds have been omitted for clarity.

The same feature is also present in the packing of the energetic nitrate salt, with the dication surrounded by trigonal-planar nitrate ions (Figure 7). In the nitrate salt, however, the dication shows the maximum deviation from planarity within the set of investigated compounds. In fact, the dihedral angle between the two pentatomic rings is 15.0(2)°. The layers of ionic/H-bonded molecules (Figure 7a,b) are parallel to the lattice plane 211, with a stacking distance of *d*_211_ = 3.32 Å.

**Figure 8:**
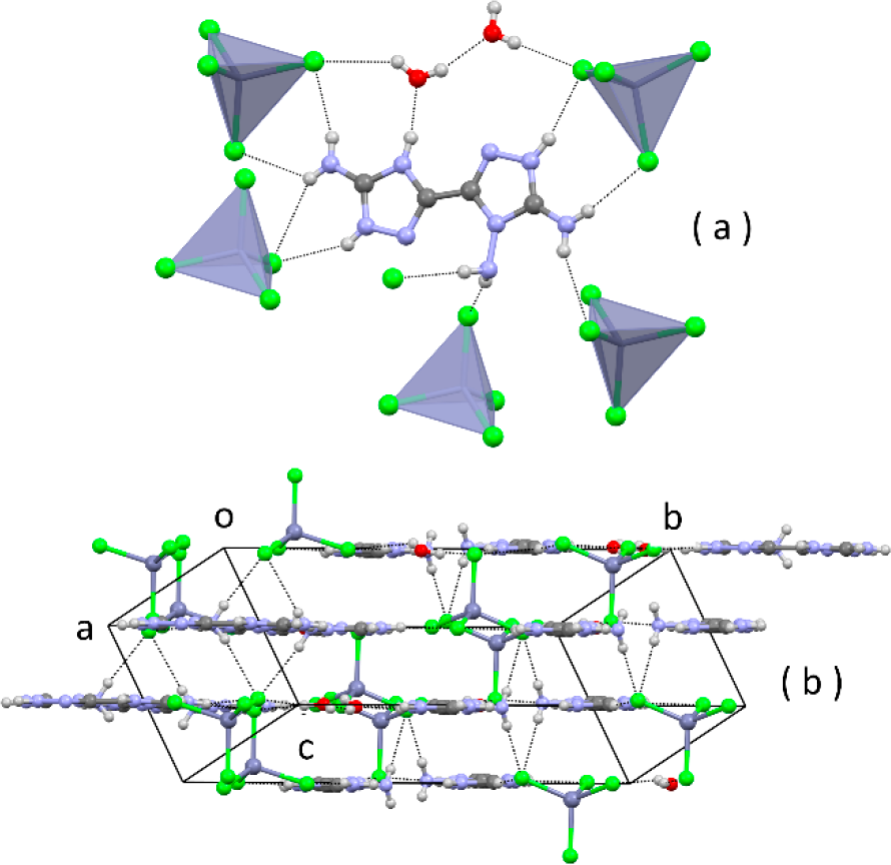
Crystal structure of (C_4_H_9_N_9_)ZnCl_4_·2H_2_O: (a) view of the cation, water molecules, and some H-bonded tetrachlorozincate anions; (b) edge view of layers of ionic/H-bonded ions. Hanging contacts have been omitted for clarity.

We have also crystallized the dication with the tetrachlorozincate complex anion (Figure 8). The crystal structure is stabilized by many N–H···Cl H bonds that are distributed over the full length of the N-rich molecule ion (Figure 8a). The layers of ionic/H-bonded molecules (Figure 8b) are parallel to the lattice plane 102, with a stacking distance of *d*_102_ = 3.16 Å.

**Figure 9:**
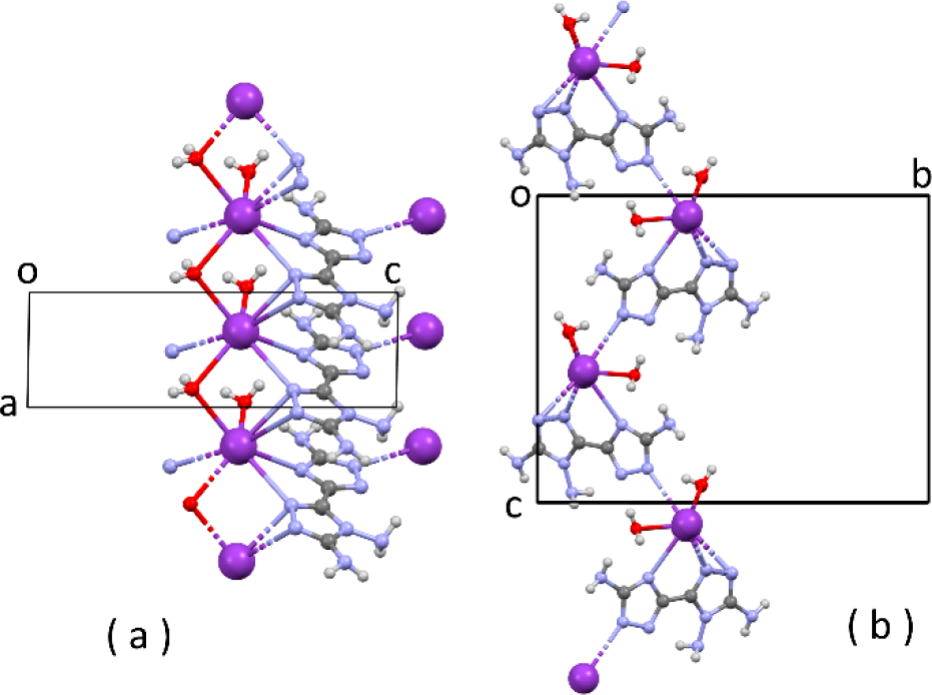
Partial packing of K(C_4_H_6_N_9_)·2H_2_O: (a) projection along *b*; (b) projection along *a*.

Thanks to the amphoteric properties of **1** (Chart 1 and Table 2), we have tried to crystallize salts of the triamine anion with inorganic cations (Na^+^, K^+^, and NH_4_^+^). As a matter of fact, neutral triamine is soluble in inorganic acids (e.g., HCl, HBr, and HClO_4_) and inorganic bases (NaOH_aq_, KOH_aq_, and aqueous ammonia). However, crystallization of neutral **1** from aqueous ammonia yielded crystals of hydrated neutral **1**. This result can be rationalized if we observe that the product of the acid constant of **1** (*K*_a3_ in Table 2) and of *K*_b_ of ammonia (1.774 × 10^–5^ at 25 °C)^20^ is almost equal to *K*_w_, and so the equilibrium constant of the reaction between ammonia and **1** is almost unitary. On the other hand, we have successfully crystallized the sodium and potassium salts of the anion. The crystal structure of K(C_4_H_6_N_9_)·2H_2_O is reported in Figure 9.

In the molecular structure of the anion, the geometry around the (C)-NH_2_ N atom is again pyramidal [the sum of the valence angles is 337(2)° and 347(2)°], and this is expected because the electron excess of the anion reduces conjugation of the (C)-NH_2_ amino groups toward the rings. The tendency toward the formation of layers is no longer observed because the packing is mainly driven by the coordination geometry of the anionic ligand to the metal ion. As is evident from Figure 9, each anion acts as a tetradentate ligand by four ring N atoms. One N atom is μ_2_ between two K^+^ ions, and a water molecule is also μ_2_-bridging between the same K^+^ ions. In this way, infinite chains running along *a* are formed by simple translation (Figure 9a). Chains are also formed running along *c*, and they are generated by the glide operation of the space group *P*2_1_/*c* (Figure 9b). Altogether, a 2D coordination network is generated.

### Characterization of Energetic Materials

**Figure 10:**
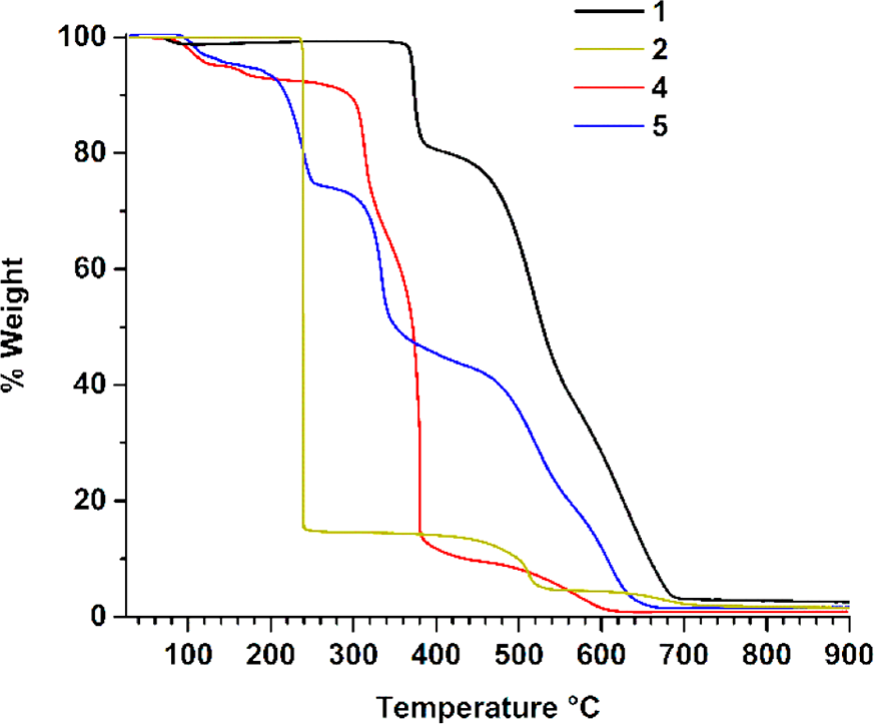
TGA of **1** and of some of its salts in air. Heating rate 10 K/min.

In Figure 10 is reported the thermogravimetric analysis (TGA) of **1** and of energetic salts of the **1** cation or dication with oxidizing anions [differential scanning calorimetry (DSC) analysis is reported in the SI].

The neutral compound shows a very remarkable thermal stability in air. After an initial loss of hydration water at about 80 °C, the anhydrous sample is stable up to 347 °C. Among similar N-rich triazoles reported in the literature,^6^**1** has the best thermal stability in air. The thermal stability of the salts of the dication (perchlorate and nitrate) is lower. The dinitramide salt, in which triamine is present as a monocation, has a very clean TGA curve: it is fully stable up to 230 °C, when it suddenly decomposes, losing 85% by weight, with an almost vertical TGA curve (green curve of Figure 10). Among the many N-rich dinitramide salts reported so far in the literature as energetic materials,^6^ the highest thermal stability is shown by FOX-12 (*N*-guanylurea dinitramide), with *T*_d_ = 215 °C.^21^ The thermal stability of nitramide **2** is significantly higher.

The impact (IS), friction (FS), and electrostatic discharge (ESD) sensitivities of **1** and some of its energetic salts were measured experimentally (grain size 100–500 μm) and are listed in Table 3. On the basis of the computed enthalpies of formation (see the SI), the detonation parameters (*V*_oD_ = velocity of detonation, *P*_C–J_ = detonation pressure, and *Q*_ex_ = heat of detonation) were calculated with the *EXPLO5* program^23^ and are also reported in Table 3.

**Table 3:** Energetic Properties and Detonation Parameters of Compounds **1**, **2**, **4**, and **5** and the Reference Compounds RDX, HAT-DN, and 5-ATN

compound	ρ^a^ (g/cm^3^)	ρ^b^ (g/cm^3^)	*T*_d_ (°C)^c^	IS (J)^d^	FS (N)^e^	ESD (J)^f^	*V*_oD_ (m/s)^g^	*P*_C–J_ (kbar)^h^	*Q*_ex_ (kJ/kg)^i^
**1**	1.629	1.599	347	>40	>360	1	7681	194	–2378
**2**	1.837	1.803	230	4	>360	0.1	8948	311	–4496
**4**	1.908	1.873	270	4	192	0.1	7033	191	–1711
**5**	1.773	1.740	210	>25	>360	0.6	8092	246	–3289
RDX^j^	1.806		204	7.5	120	0.20	8861	345	–5845
HAT-DN^k^	1.856		117	2	20	0.75	9429	384	6186
5-ATN^k^		1.807	190	10	>324		8898	357	–4603

a Cystallographic density at −100 °C.

b Calculated density at 298 K, according to ref (22).

c Decomposition temperature (onset) for the anhydrous sample in the TGA runs of Figure 10.

d Impact sensitivity (BAM drop hammer).

e Friction sensitivity (BAM friction tester).

f Electrostatic discharge sensitivity.

g Calculated detonation velocity.

h Calculated detonation pressure.

i Calculated heat of detonation.

j Data taken from ref (6).

k Data taken from ref (2).

Since the development of RDX (1,3,5-trinitro-1,3,5-triazacyclohexane or cyclonite), any newly synthesized energetic compounds must face RDX, particularly in terms of the detonation pressure and detonation velocity, which are very important parameters in secondary explosives. For these reasons, the energetic properties of RDX have been added in Table 3 for a direct comparison. We have also added to Table 3 the literature data^2^ for two reference energetic salts containing inorganic anions: 5-aminotetrazolium dinitramide (HAT-DN) and 5-aminotetrazolium nitrate (5-ATN). Energetic compounds should also be stable with respect to the temperature, have a high density, be safe to handle, and be cheap to synthesize. In terms of the thermal stability, both **1** and **2** surpass the 200 °C benchmark and so largely outperform RDX, as well as HAT-DN and 5-ATN. **2** slightly outperforms RDX and 5-ATN also in the detonation velocity, while its performances in terms of the detonation pressure are slightly lower. Concerning the experimentally determined sensitivities, **1** is insensitive to both impact and friction. **2** is impact-sensitive, with a measured value in the range observed for other N-rich dinitramides: ammonium dinitramide (ADN) 5 J, triaminoguanidinium dinitramide (TAGDN) 2 J,^6^ and HAT-DN. On the other hand, **2** is friction-insensitive, a significant result, inasmuch as other N-rich dinitramides, including HAT-DN, have high sensitivity toward friction (ADN 72 N and TAGDN 24 N).^6^**4** is impact-sensitive and moderately sensitive to friction, while **5** is less impact-sensitive than **2** and **4** and friction-insensitive, and so it is better performing than nitrate 5-ATN. The lower impact sensitivity of **5**, compared with **2** and **4**, can be related to some of the structural features discussed above. In **5**, the dication shows the highest deviation from coplanarity of the two rings, and this produces an increase of the stacking distance between the planes of ionic/H-bonded molecules and a reduced density. Nitramide **2** can be considered to be the most interesting energetic material within the set investigated, although its impact sensitiveness hinders its use as main explosive. Altogether, the salt compounds described here could be of potential interest as propellant charges, as additives in propellant charges, or as gas generators.

## Conclusion

We have presented the N-rich bis(triazole) compound **1** and have investigated its acid–base behavior. An interesting feature of **1** is the existence of well-defined pH intervals in which it is present in solution as neutral, singly protonated, doubly protonated, and deprotonated forms. This discloses the possibility of selective crystallization from a solution of salts containing different ionic forms of **1**, which we have experimentally realized. In fact, salts containing all of the ionic species of **1** were crystallized and structurally characterized by X-ray analysis. For some salts containing energetic counterions (nitrate, perchlorate, and dinitramide), the sensitivities were experimentally determined, and the detonation parameters were computed.

Our present study paves the way to more specific studies of energetic materials based on **1**. Because of the existence of two different protonated forms, the set of possible energetic salts of **1** to be investigated is very large in principle. As an example, further studies could be directed to a comparison of the properties of energetic salts containing the same counterion but different protonated forms of **1**.

## Experimental Part

***Caution!** The compounds in this work are potentially energetic materials that could explode under certain conditions (such as impact, friction, or electric discharge). Experiments should be performed on a small scale. Appropriate safety precautions, including the use of safety shields and personal protection (safety glasses, ear plugs, and gloves), are suggested at all times during handling of these compounds.*

### General Procedures

All reagents were of analytical grade and were used without further purification. Melting points were determined by temperature-controlled optical microscopy (Zeiss Axioskop polarizing microscope equipped with a Linkam PR600 heating stage). TGA was performed with a PerkinElmer TGA 4000 apparatus. DSC analysis was performed with a PerkinElmer Pyris instrument. NMR spectra were recorded with a Bruker spectrometer operating at 400 MHz, in deuterated dimethyl sulfoxide (DMSO-*d*_6_). Electrospray ionization (ESI) mass spectrometry (MS) analyses were recorded with an Applied Biosystems API 2000 mass spectrometer equipped with an electrospray source used in the positive mode. Elemental analyses were performed using a FlashEA 1112 analyzer (Thermo Fisher Scientific Inc.) and a Netsch STA 429 apparatus.

#### Synthesis of **1**

Commercial 5-amino-1*H*-1,2,4-triazole-3-carboxylic acid (5.00 g, 4.90 × 10^–2^ mol) and diaminoguanidine monohydrochloride (6.40 g, 5.09 × 10^–2^ mol, 30% excess by mol) were finely ground in a mortar. The mixture was added in portions, under mechanical stirring, to a beaker containing poly(phosphoric acid) (40 g) at 100 °C (Scheme S1). After a few minutes, the evolution of gaseous HCl was observed from the reaction mixture. The temperature of the pasty reaction mixture was increased to 150 °C, and the mixture reacted for 5 h under stirring. Afterward, the mixture was poured into cold water (100 mL), and the pH of the resulting solution was increased to 5 by the addition of a concentrated solution of NaOH. Raw **1**, in the form of an off-white solid, was obtained, filtered, washed with cold water, and dried in an oven at 100 °C. Raw **1** (5.5 g) was suspended in water (100 mL). Concentrated HCl (37%; 20 mL) was added drop by drop under stirring, and the suspension was heated. Upon addition of the acid and heating, the suspension progressively became a clear, pale-brown solution. The solution was kept boiling under stirring until the volume reduced 50 mL. Then it was cooled to room temperature, and a white solid (the dichlorhydrate salt) formed. The solid was recovered by filtration and washed on the filter with ethanol. Then it was dried in an oven at 110 °C. A total of 4.9 g of the dichlorhydrate salt was obtained. The salt product was solved in hot water (about 100 mL). A 1 M solution of KOH was added drop by drop until the pH was 6–7. A crystalline precipitate formed. The suspension was cooled to room temperature, and the precipitate was filtered, washed with water on the filter, and dried in an oven at 110 °C overnight. In this way, 3.98 g of pure **1** was obtained. Yield: 3.98 g (45%). Mp: 347 °C (dec). ^1^H NMR (400 MHz, DMSO-*d*_6_): δ 5.67 (s, 2H), 5.77 (s, 2H), 6.18 (s, 2H) 12.33 (s, 1H). ^13^C NMR (100 MHz, DMSO-*d*_6_): δ 142.59, 150.58 155.47 157.24. MS (ESI, positive mode). Calcd for C_4_H_7_N_9_: *m*/*z* 181.16. Found: *m*/*z* 182.0 (M^+^·H). Anal. Calcd for C_4_H_7_N_9_·0.5H_2_O: C, 25.26; H, 4.24; N, 66.29. Found: C, 25.86; H, 3.69; N, 67.00.

#### Synthesis of (C_4_H_8_N_9_)(N_3_O_4_)

(C_4_H_9_N_9_)(ClO_4_)_2_ (0.667 g, 1.75 mmol) was dissolved in 25 mL of hot water. The solution was added to another solution containing KN_3_O_4_ (0.508 g, 3.50 mmol) and 2 mL of water. Pale-pink prismatic crystals of the triamine monocation salt were obtained by slow cooling to room temperature from a warm water bath (70 °C) with quantitative yield. Anal. Calcd for (C_4_H_8_N_9_)(N_3_O_4_): C, 16.66; H, 2.80; N, 58.32. Found: C, 16.31; H, 2.43; N, 58.81.

#### Synthesis of (C_4_H_9_N_9_)Br_2_

A total of 10 drops of a HBr concentrated solution (48%, v/v) was added to a hot water solution of 30 mg of **1** (0.150 mmol). Prismatic colorless crystals of the dihydrate bromide salt were obtained by slow solvent evaporation at room temperature in 2 days, with 86% yield. Anal. Calcd for (C_4_H_9_N_9_)Br_2_·2H_2_O: C, 12.67; H, 3.46; N, 33.26. Found: C, 12.42; H, 3.31; N, 33.69.

#### Synthesis of (C_4_H_9_N_9_)(ClO_4_)_2_

A total of 5 drops of a HClO_4_ concentrated solution (70%, v/v) was added to a hot water solution of 30 mg of **1** (0.150 mmol). Prismatic colorless crystals of the perchlorate salt hydrate were obtained by slow evaporation at room temperature in 2 days, with 90% yield. The sample was dehydrated by keeping it in a desiccator over CaCl_2_ for 1 week. Anal. Calcd for C_4_H_9_N_9_(ClO_4_)_2_: C, 12.57; H, 2.37; N, 32.99. Found: C, 12.51; H, 2.64; N, 32.38.

#### Synthesis of (C_4_H_9_N_9_)(NO_3_)_2_

A total of 5 drops of a HNO_3_ concentrated solution (65%, v/v) was added to a hot water solution of 30 mg of **1** (0.150 mmol). Pale-pink lozenge crystals of the nitrate salt dihydrate was obtained by slow cooling to room temperature from a warm water bath (70 °C) with quantitative yield. Anal. Calcd for (C_4_H_9_N_9_)(NO_3_)_2_·H_2_O: C, 14.77; H, 3.41; N, 47.37. Found: C, 14.99; H, 3.75; N, 46.98.

#### Synthesis of (C_4_H_9_N_9_)(ZnCl_4_)

**1** (30 mg, 0.150 mmol) was dissolved in 1 mL of hot water. The solution was added to another solution containing ZnCl_2_ (20 mg, 0.150 mmol), water (1 mL), and 10 drops of a concentrated HCl solution (37%, v/v). The solution was left undisturbed, and pale-brown prismatic crystals were obtained in 2 days, with 60% yield. Anal. Calcd for (C_4_H_9_N_9_)ZnCl_4_·2H_2_O: C, 11.24; H, 3.06; N, 29.49. Found: C, 11.71; H, 2.83; N, 28.97.

#### Synthesis of K(C_4_H_6_N_9_)

Brown prismatic crystals of the K(C_4_H_6_N_9_) dihydrate salt were grown in 1 day by slow cooling to room temperature of a hot water solution containing 200 mg of **1** (1 mmol) and a KOH concentrated solution (2 mL) with quantitative yield. Anal. Calcd for K(C_4_H_6_N_9_)·2H_2_O: C, 18.82; H, 3.95; N, 49.38. Found: C, 18.23; H, 4.42; N, 49.27.

### Computational Details

Quantum-chemical computations were carried with the *Gaussian 16* package by using density functional theory (DFT).^24^ The B3LYP functional was employed throughout in conjunction with the 6-31+G** basis set. B3LYP has proven to give excellent performance, nearly reproducing experimental electrical and optical properties for organic molecules.^25,26^ Solvent (water) effects were included by the polarizable continuum model.^27^ The nature of the located stationary points was verified by checking the eigenvalues of the Hessian matrix; all of the minimum-energy structures have positive eigenvalues. For all tautomers of Chart 2, a molecular-mechanics scan based on the *Spartan* program was performed, in order to find possible conformers.

### Acid–Base Equilibria

The protolytic equilibria of **1** were studied by UV–vis absorption spectroscopy in 0.5 M NaCl, as the ionic medium, following a procedure already described^8,18^ and detailed in the SI. The experiments were performed as acid–base titrations at a constant total concentration of **1** (*c* = 5.01 × 10^–5^ M). The investigated pH range extends from 0.3 to 12. For each experimental point, the equilibrium free proton concentration was evaluated from the measured electromotive force at the ends of the galvanic cell GE/TS/RE, where TS indicates the test solution, GE is the glass electrode, and RE is a reference electrode [0.5 M NaCl|Hg_2_Cl_2_|Hg (Pt)] placed outside but electrically connected to TS through a salt bridge. All of the experiments were carried out in air, in a thermostat at 25.00 ± 0.03 °C. Absorption spectra were recorded with a Varian Cary 50 UV–vis spectrophotometer using a 1 cm cell. The primary spectrophotometric data (*A*, pH, and λ) were elaborated graphically^28^ and numerically by using the *HYPSPEC 2008* program^29^ for determination of the equilibrium constants in solution.

### X-ray Analysis

All data for crystal structure determinations were measured on a Bruker-Nonius Kappa CCD diffractometer equipped with an Oxford Cryostream 700 apparatus, using graphite-monochromated Mo Kα radiation (λ = 0.71073 Å). Reduction of data and semiempirical absorption correction were done using the *SADABS* program.^30^ The structures were solved by direct methods (*SIR97* program^31^) and refined by the full-matrix least-squares method on *F*^2^ using the *SHELXL-2015* program^32^ with the aid of the program *WinGX*.^33^ H atoms bonded to N atoms, which are essential in the identification of tautomers, and those bonded to O atoms in water molecules, were clearly found in difference Fourier maps as the first maxima, and in some cases, their coordinates were refined. For all H atoms, *U*_iso_ = 1.2*U*_eq_ of the carrier atom was assumed. Full crystal and refinement data are summarized in Tables S1 and S2. Analysis of the crystal packing was performed using the program *Mercury*.^34^ CCDC 2092331, 2092333, 2092335, 2092337, 2092339, 2092341, and 2092342 contain the supplementary crystallographic data for this paper (see the SI).

### Sensitivity Testing

The impact sensitivity tests were carried out according to *STANAG 4489*(35) modified instruction^36^ using a BAM (Bundesanstalt für Materialforschung) drophammer.^37^ The friction sensitivity tests were carried out according to *STANAG 4487*(38) modified instruction^39^ using the BAM friction tester. The classification of the tested compounds results from the “UN Recommendations on the Transport of Dangerous Goods”.^40^ Additionally, all compounds were tested on the sensitivity toward electrical discharge using the Electric Spark Tester ESD 2010 EN.^41^

## Supporting Information Available

Scheme of the synthesis of **1**, ^1^H and ^13^C NMR and mass spectra of **1**, DSC thermograms of **1** and its energetic salts, detailed description of the UV–vis and electrochemical methods used in the study of the acid–base properties, full crystallographic details, full details of the computation of the energetic properties, and geometry of DFT-optimized tautomers/conformers (PDF)

## Accession Codes

CCDC 2092331, 2092333, 2092335, 2092337, 2092339, 2092341, and2092342 contain the supplementary crystallographic data for this paper. These data can be obtained free of charge via www.ccdc.cam.ac.uk/data_request/cif, or by emailing data_request@ccdc.cam.ac.uk, or by contacting The Cambridge Crystallographic Data Centre, 12 Union Road, Cambridge CB2 1EZ, UK; fax: +44 1223 336033.
