# Supplementary material for: High-Energy-Density Materials: An Amphoteric N-Rich Bis(triazole) and Salts of Its Cationic and Anionic Species
Source: Inorg Chem. 2021 Oct 12;60(21):16213–22. doi: 10.1021/acs.inorgchem.1c02002 (PMC8564754; doi:10.1021/acs.inorgchem.1c02002)
Supplement: Supplementary file 1 — ic1c02002_si_001.pdf [file ic1c02002_si_001.pdf]

High energy density materials: an amphoteric N-rich bistriazole and salts of its cationic and anionic species

Emmanuele Parisi,[1] Alessandro Landi,[2] Sandra Fusco,[1] Carla Manfredi,[1] Andrea Peluso,[2] Sabrina Wahler,[3] Thomas M. Klapötke,[3] Roberto Centore*[1]

[1]Department of Chemical Sciences, University of Naples Federico II, Via Cinthia, I-80126 Naples, Italy

[2]Department of Chemistry and Biology, University of Salerno, Via Giovanni Paolo II, 132, I-84084 Fisciano, Saler-no, Italy

[3]Department of Chemistry, Energetic Materials Research, Ludwig-Maximilian University, Butenandtstr. 5 - 13 (D), D-81377 Münich, Germany

Supporting Information (SI)

Index

Synthetic scheme of **1**……………………………………………………..p. 2

NMR and mass spectra of **1**………………………………………………p. 3

Acid-base equilibria, UV-VIS spectra, and titrations………..…………...p. 6

Crystal, collection and refinement data…………………………………..p. 10

DSC analysis……………………………………………………………...p. 12

Computational part (energetics)…………………………………………p. 13

Optimized Structures of tautomers/conformers…………………………p. 18

References………………………………………………………………..p. 32

Synthetic Scheme of 1

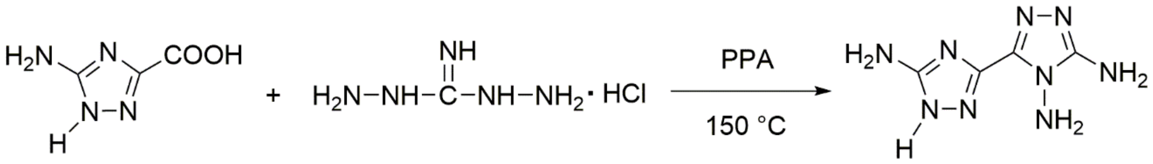

Scheme S1. Synthesis of **1**.

NMR and mass spectra of 1

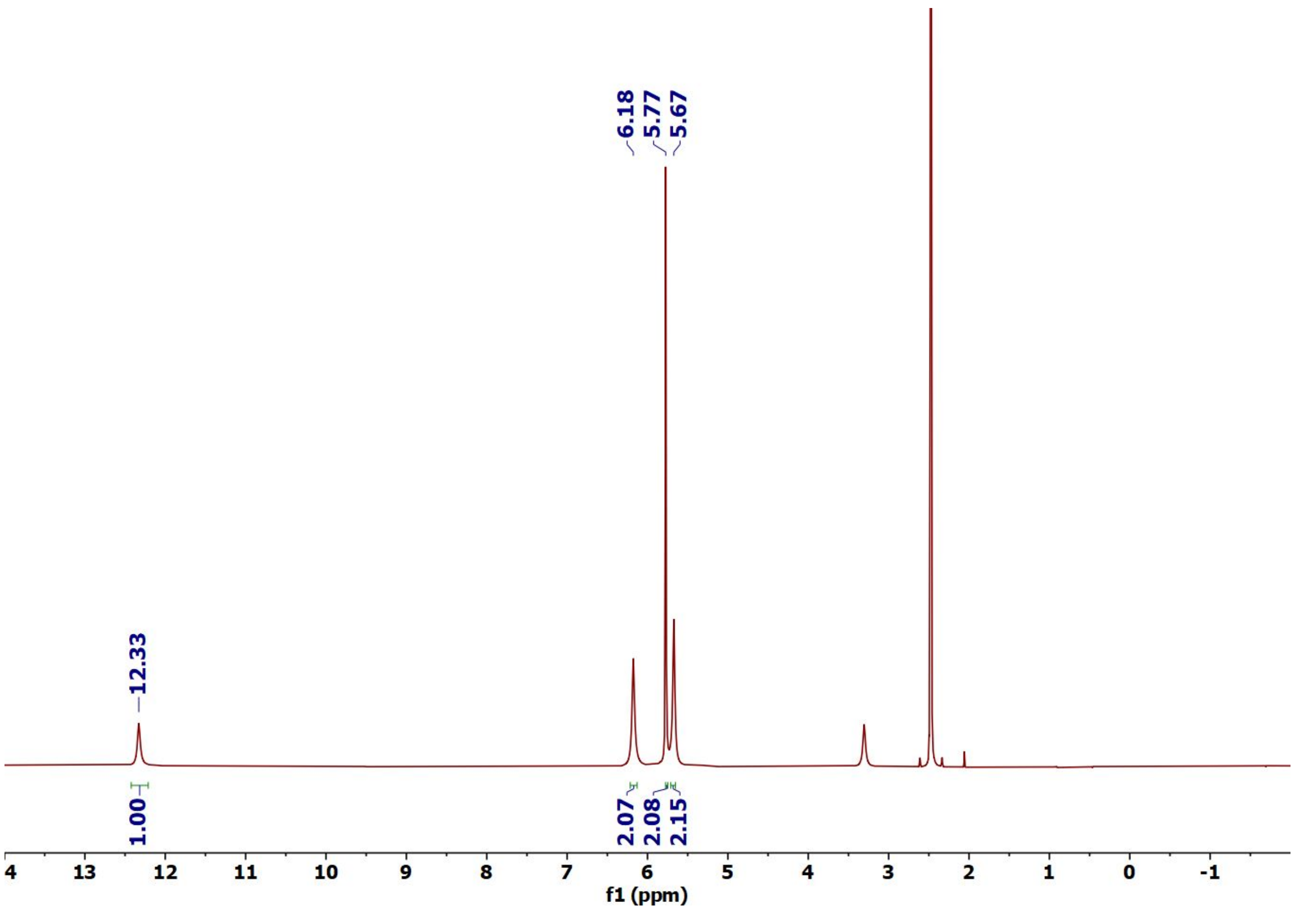

Fig. S1. ^{1}H-NMR spectrum of **1** in DMSO-d6 at 25 °C. The signals at 2.51 ppm and at 3.35 ppm are due to the solvent and water, respectively.

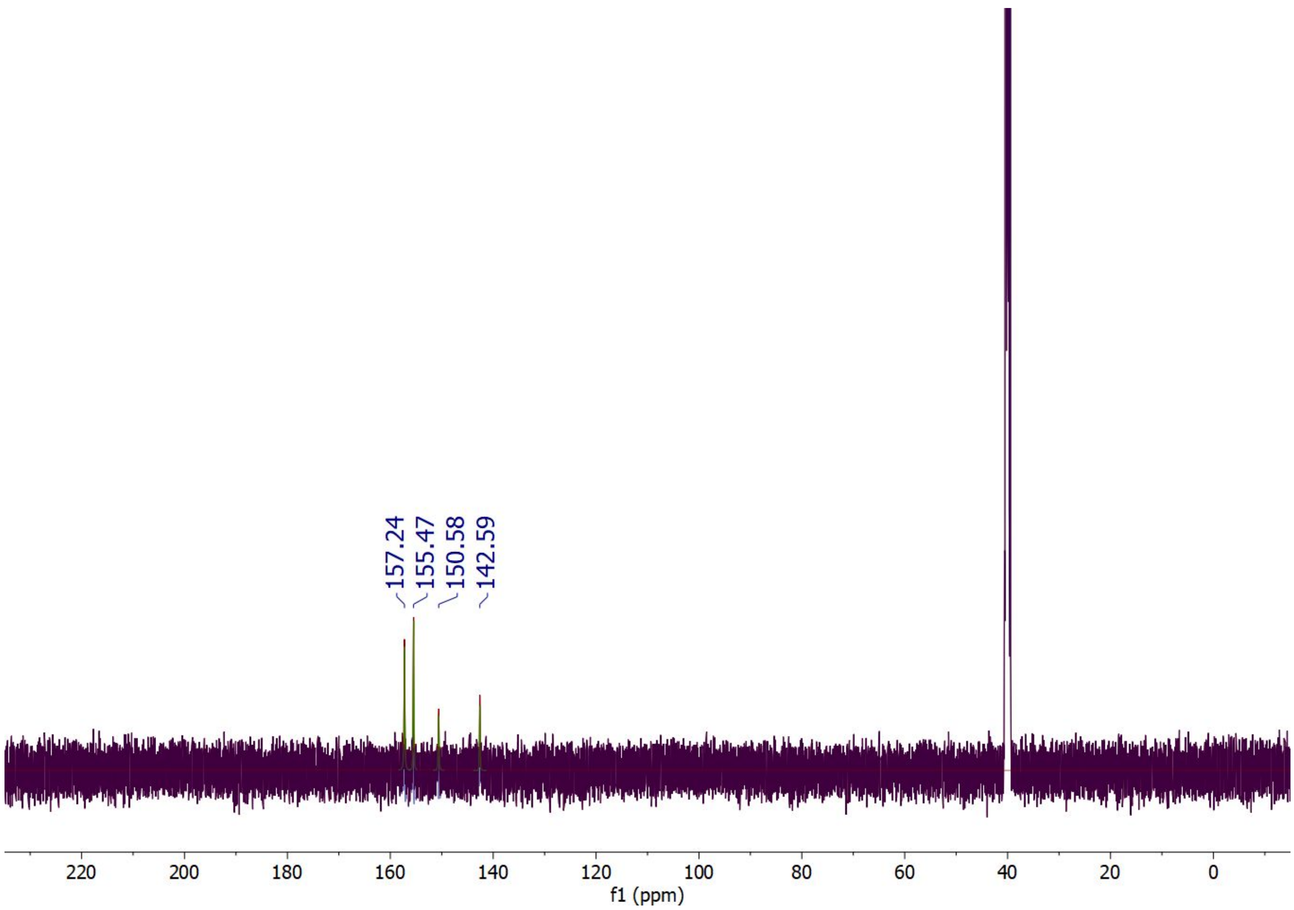

Fig. S2. ^{13}C-NMR spectrum of **1** in DMSO-d6, at 25 °C. The signals nearly 40 ppm are due to the solvent.

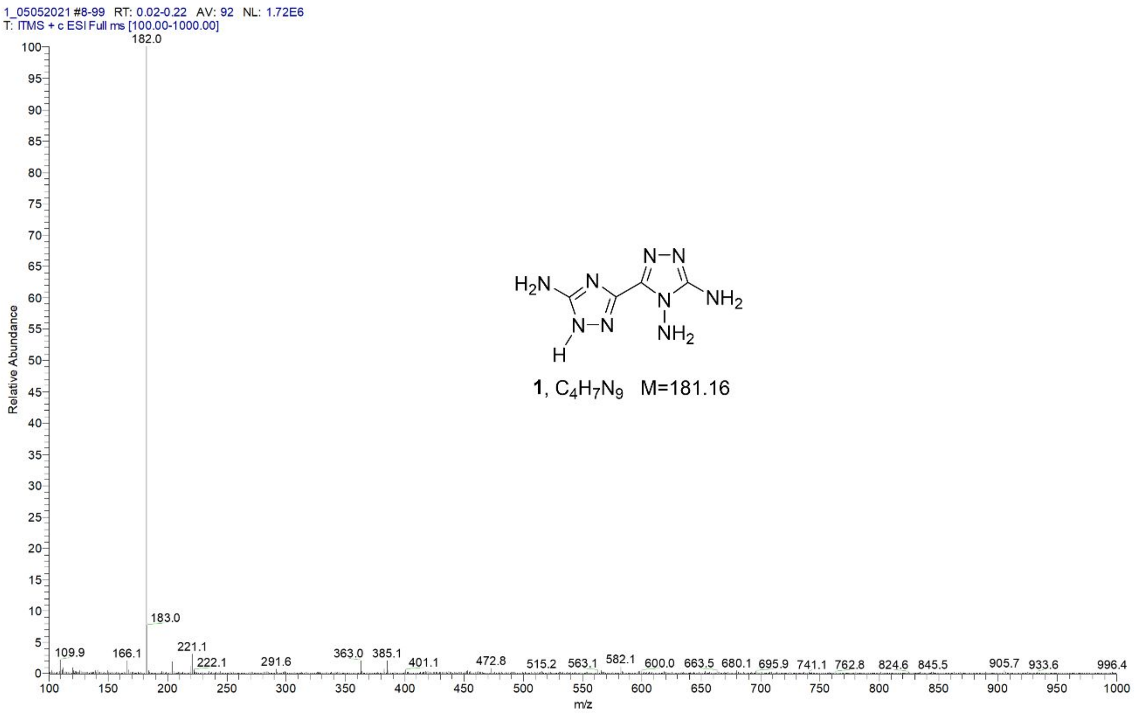

Fig. S3. ESI mass spectrum of **1**.

Acid-base equilibria, UV-VIS spectra, and UV-VIS titrations

In this section, **1** will also be indicated as *HL*, the singly protonated form of **1** as H_2L^+, the doubly protonated form as H_3L^{++}, and the deprotonated form as L^-. The protolytic equilibria of **1** were studied by UV-VIS absorption spectroscopy in 0.5 M NaCl, as the ionic medium. The **1** stock solutions were prepared starting from the solid. Deionized and doubly distilled water was used to prepare all aqueous solutions. NaCl (Fluka, dried overnight at 120 °C) was used to prepare the ionic medium solutions. Stock solutions of HCl (Carlo Erba p.a.) were standardized against $KHCO_3$ using methyl red as indicator, with a reproducibility of 0.1 %. NaOH (Baker p.a.) stock solutions were standardized against standardized HCl solutions. The experiments were performed as acid-base titrations at constant total concentration of **1** (C= $5.01 \cdot 10^{-5}$ M). The investigated pH spans between 0.7 and 10. For each experimental point at 2.5<pH<10, the equilibrium free proton concentration was evaluated from the measured electromotive force at the ends of the galvanic cell GE/TS/RE, where TS indicates the Test solution, GE is the glass electrode and RE is a reference electrode (0.5 M NaCl|Hg_2Cl_2|Hg(Pt)) placed outside but electrically connected to TS through a salt bridge. The Nernst potential of the cell, E(mV), can be written as $E = E_0 + 59.16 \log [H^+] + E_j$ where E_j= liquid junction potential due to the replacement of Na^+ with H^+.[1] The evaluation of the constant of the glass electrode, E_0, was performed in the first stage of each experiment by a coulometric titration, using the Gran method.[2] The test solutions at 0.3<pH<2 were obtained analytically. All the experiments were carried out in air in a thermostat, at 25.00 ± 0.03 °C. Potentiometric experimental data were collected by means of an automatic data acquisition system based on Hewlett-Packard (HP) instrumentation. The glass membrane electrodes reversible to protons were supplied by Metrohm. Highly precise (± 0.02 mV) emf measurements were made by adapting the impedance of the glass electrode through operational amplifiers. Coulometric variations of the solution composition were carried out using a Hewlett Packard "DC Power Supply". The intensity of the current in the electrolysis circuit was measured from the potential drop at the ends of a calibrated resistance; the current density was set at about 1 mA/cm^2. Absorption spectra were recorded with a Varian Cary 50 UV-Vis spectrophotometer using 1 cm cell. The primary spectrophotometric data (Absorbance, pH, λ) were elaborated both graphically[3] and numerically, by the HYPERQUAD program.[4] In Figure S5, the continuous curves were obtained by fitting the experimental points with an equation that relates the Absorbance of the solution (at a given wavelength) with the pH, the analytical concentration of **1**, and the equilibrium constants, by assuming the law of additivity of absorbances (Bouger-Lambert-Beer equation). The Bouger-Lambert-Beer equation is now explicitly derived for the system **1** (HL).

From the Lambert-Beer law, assuming additivity of the absorbances, it is

$$A^{\lambda} = b\left(\sum_i \varepsilon_i^{\lambda} c_i\right) = b\left(\varepsilon_1^{\lambda}[H_3L^{++}] + \varepsilon_2^{\lambda}[H_2L^{+}] + \varepsilon_3^{\lambda}[HL] + \varepsilon_4^{\lambda}[L^{-}]\right) \quad (1)$$

in which *b* is the optical path of the cell (1 cm) and ε_i^{λ} is the molar extinction coefficient of the species *i* at the given wavelength. The analytical concentration of **1** (*HL*), *C*, is given by

$$C = [H_3L^{++}] + [H_2L^{+}] + [HL] + [L^{-}] \quad (2)$$

The acid-base conditional equilibrium constants are

$$K_{a1} = \frac{[H_2L^{+}]\cdot[H_3O^{+}]}{[H_3L^{++}]}, K_{a2} = \frac{[HL]\cdot[H_3O^{+}]}{[H_2L^{+}]}, K_{a3} = \frac{[L^{-}]\cdot[H_3O^{+}]}{[HL]} \quad (3)$$

From these, it is possible to express the concentration of all species but one (e. g. the neutral one), as a function of the equilibrium constants and the concentration of H_3O^{+}:

$$[H_3L^{++}] = \frac{[H_2L^{+}]\cdot[H_3O^{+}]}{K_{a1}}, [H_2L^{+}] = \frac{[HL]\cdot[H_3O^{+}]}{K_{a2}}, [L^{-}] = \frac{[HL]\cdot K_{a3}}{[H_3O^{+}]}$$

that is

$$[H_3L^{++}] = \frac{[HL]\cdot[H_3O^{+}]^2}{K_{a1}\cdot K_{a2}}, [H_2L^{+}] = \frac{[HL]\cdot[H_3O^{+}]}{K_{a2}}, [L^{-}] = \frac{[HL]\cdot K_{a3}}{[H_3O^{+}]} \quad (4)$$

Putting (4) in (2) it is

$$C = \frac{[HL]\cdot[H_3O^{+}]^2}{K_{a1}\cdot K_{a2}} + \frac{[HL]\cdot[H_3O^{+}]}{K_{a2}} + [HL] + \frac{[HL]\cdot K_{a3}}{[H_3O^{+}]} \quad (5)$$

So, it is

$$[HL] = \frac{CK_{a1}K_{a2}[H_3O^{+}]}{[H_3O^{+}]^3 + K_{a1}[H_3O^{+}]^2 + K_{a1}K_{a2}[H_3O^{+}] + K_{a1}K_{a2}K_{a3}} \quad (6)$$

Putting (4) and (6) in (1) yields

$$A = b\cdot\left(\varepsilon_1^{\lambda}\frac{[HL]\cdot[H_3O^{+}]^2}{K_{a1}K_{a2}} + \varepsilon_2^{\lambda}\frac{[HL]\cdot[H_3O^{+}]}{K_{a2}} + \varepsilon_3^{\lambda}[HL] + \varepsilon_4^{\lambda}\frac{[HL]\cdot K_{a3}}{[H_3O^{+}]}\right) \quad (7)$$

$$A = b\cdot[HL]\left(\varepsilon_1^{\lambda}\frac{[H_3O^{+}]^2}{K_{a1}K_{a2}} + \varepsilon_2^{\lambda}\frac{[H_3O^{+}]}{K_{a2}} + \varepsilon_3^{\lambda} + \varepsilon_4^{\lambda}\frac{K_{a3}}{[H_3O^{+}]}\right) \quad (8)$$

$$A = b \cdot \frac{C K_{a1} K_{a2} [H_3O^+]}{[H_3O^+]^3 + K_{a1}[H_3O^+]^2 + K_{a1}K_{a2}[H_3O^+] + K_{a1}K_{a2}K_{a3}} \left(\varepsilon_1^{\lambda} \frac{[H_3O^+]^2}{K_{a1}K_{a2}} + \varepsilon_2^{\lambda} \frac{[H_3O^+]}{K_{a2}} + \varepsilon_3^{\lambda} + \varepsilon_4^{\lambda} \frac{K_{a3}}{[H_3O^+]} \right) \quad (9)$$

$$A = b \cdot \frac{C}{[H_3O^+]^3 + K_{a1}[H_3O^+]^2 + K_{a1}K_{a2}[H_3O^+] + K_{a1}K_{a2}K_{a3}} \left(\varepsilon_1^{\lambda}[H_3O^+]^3 + \varepsilon_2^{\lambda} K_{a1}[H_3O^+]^2 + \varepsilon_3^{\lambda} K_{a1}K_{a2}[H_3O^+] + \varepsilon_4^{\lambda} K_{a1}K_{a2}K_{a3} \right) \quad (10)$$

and, finally, the Bouger-Lambert-Beer equation

$$A = b \cdot \frac{C}{10^{-3pH} + 10^{-2pH - pK_{a1}} + 10^{-pH - pK_{a1} - pK_{a2}} + 10^{-pK_{a1} - pK_{a2} - pK_{a3}}} \left(\varepsilon_1^{\lambda} 10^{-3pH} + \varepsilon_2^{\lambda} 10^{-2pH - pK_{a1}} + \varepsilon_3^{\lambda} 10^{-pH - pK_{a1} - pK_{a2}} + \varepsilon_4^{\lambda} 10^{-pK_{a1} - pK_{a2} - pK_{a3}} \right)$$

The UV-VIS spectra, at constant total concentration and different pH, are reported in Fig. S4 for **1**.

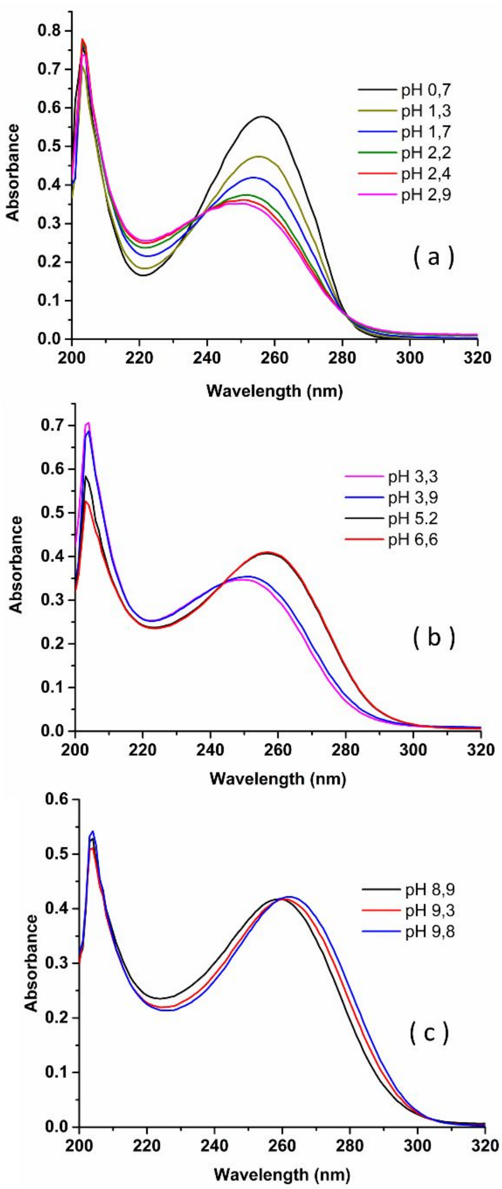

Fig. S4. UV−VIS absorption spectra of **1** at constant total concentration, C = 5.01 × 10^{-5} M, in NaCl 0.5 M recorded at $0.6 \leq pH \leq 9.9$. The spectra have been grouped in three set of curves for easier lecture.

The plots of Absorbance *vs* pH at fixed wavelength and the fitting of the points using the Bouger-Lambert-Beer equation are reported in Fig. S5.

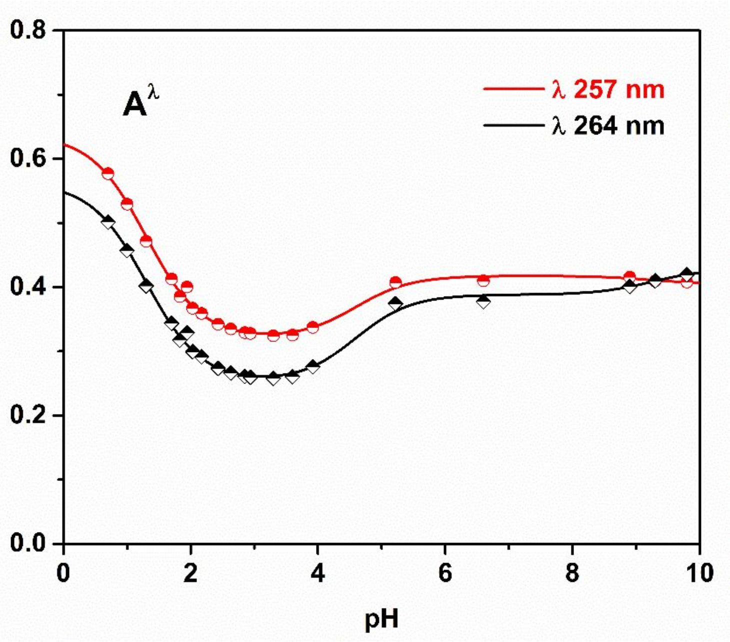

Fig. S5. UV-VIS titration curves of **1**, at two different wavelengths. The continuous curves have been constructed by fitting the experimental points (absorbance *vs* pH data taken from Fig. S4) with the Bouger-Lambert-Beer equation.

Based on the distribution diagram (Fig. 2 of the typescript), the isosbestic points at λ=282 nm in Fig. S4(a), at 244 nm in Fig. S4(b), and at 259 nm in Fig. S4(c), are related, respectively, with the three equilibria

$$H_3L^{2+} + H_2O \rightleftarrows H_3O^+ + H_2L^+$$

$$H_2L^+ + H_2O \rightleftarrows H_3O^+ + HL$$

$$HL + H_2O \rightleftarrows H_3O^+ + L^-$$

Tab. S1. Crystal, collection, and refinement data for the structures described in the paper.

	**1**	**2**	**3**	**4**
Chemical formula	$C_4H_7N_9\cdot 0.5(H_2O)$	$C_4H_8N_9(N_3O_4)$	$(C_4H_9N_9)Br_2\cdot 2H_2O$	$(C_4H_9N_9)(ClO_4)_2\cdot H_2O$
M_r	190.19	288.22	379.05	400.12
System	Triclinic	Triclinic	Triclinic	Triclinic
Space group	*P*-1	*P*-1	*P*-1	*P*1
Temp. (K)	173	173	173	173
a (Å)	7.1560(7)	7.722(2)	6.5350(15)	5.2730(8)
b (Å)	11.6750(16)	8.773(2)	7.0520(19)	7.239(3)
c (Å)	19.209(3)	9.442(4)	14.486(6)	9.730(3)
α (°)	85.50(2)	64.422(15)	96.67(4)	100.17(3)
β (°)	85.093(14)	66.67(2)	100.35(3)	95.68(2)
γ (°)	76.354(11)	71.33(3)	106.565(16)	105.38(3)
V (Å^3)	1551.0(4)	521.1(3)	619.5(4)	348.29(18)
Z	8	2	2	1
λ	Mo $K\alpha$	Mo $K\alpha$	Mo $K\alpha$	Mo $K\alpha$
μ (mm^{-1})	0.13	0.16	6.55	0.54
Cryst. size (mm)	0.45 × 0.40 × 0.30	0.20 × 0.15 × 0.15	0.50 × 0.40 × 0.30	0.40 × 0.25 × 0.15
Meas., ind. reflns	22895, 7082	6676, 2386	8954, 2789	4548, 2585
R_{int}	0.045	0.078	0.035	0.026
$(\sin \theta/\lambda)_{max}$ (Å^{-1})	0.650	0.650	0.650	0.649
$R[I > 2\sigma(I)]$, wR(all), S	0.046, 0.123, 1.05	0.059, 0.162, 1.04	0.026, 0.062, 1.10	0.025, 0.067, 1.06
Data	7082	2386	2789	2585
Parameters	593	205	193	250
Restraints	6	0	0	3
$\Delta\rho_{max}$, $\Delta\rho_{min}$ (e Å^{-3})	0.64, −0.30	0.34, −0.32	0.45, −0.64	0.22, −0.41
CCDC	2092331	2092333	2092335	2092337

Tab. S2. Crystal, collection, and refinement data for the structures described in the paper.			
	**5**	**6**	**7**
Chemical formula	$(C_4H_9N_9)(NO_3)_2\cdot H_2O$	$(C_4H_9N_9)ZnCl_4\cdot 2H_2O$	$K(C_4H_6N_9)\cdot 2H_2O$
M_r	325.24	427.41	255.31
System	Triclinic	Monoclinic	Monoclinic
Space group	*P*-1	$P2_1/c$	$P2_1/c$
Temperature (K)	173	173	173
a (Å)	6.690(5)	9.0580(15)	4.230(3)
b (Å)	7.290(4)	21.642(3)	17.330(5)
c (Å)	13.985(5)	8.4120(19)	13.579(4)
α (°)	75.21(10)	90	90
β (°)	81.90(10)	110.89(2)	90.99(2)
γ (°)	67.68(9)	90	90
V (Å^3)	609.3(8)	1540.7(5)	995.3(8)
Z	2	4	4
λ	Mo *K*α	Mo *K*α	Mo *K*α
μ (mm^{-1})	0.16	2.30	0.54
Cryst. size (mm)	0.45 × 0.35 × 0.30	0.20 × 0.20 × 0.20	0.45 × 0.20 × 0.20
Meas., ind. reflns	7338, 2778	13171, 3507	6339, 2241
R_{int}	0.036	0.021	0.029
$(\sin\theta/\lambda)_{max}$ (Å^{-1})	0.649	0.650	0.650
$R[I > 2\sigma(I)]$, wR(all), S	0.038, 0.097, 1.07	0.024, 0.052, 1.14	0.031, 0.078, 1.06
Reflections	2778	3507	2241
Parameters	232	200	175
Restraints	0	10	0
$\Delta\rho_{max}$, $\Delta\rho_{min}$ (e Å^{-3})	0.29, −0.30	0.29, −0.33	0.28, −0.27
CCDC	2092339	2092341	2092342

DSC Analysis

The DSC curves of energetic compounds **1**, **2**, **4** and **5** are reported in Fig. S6.

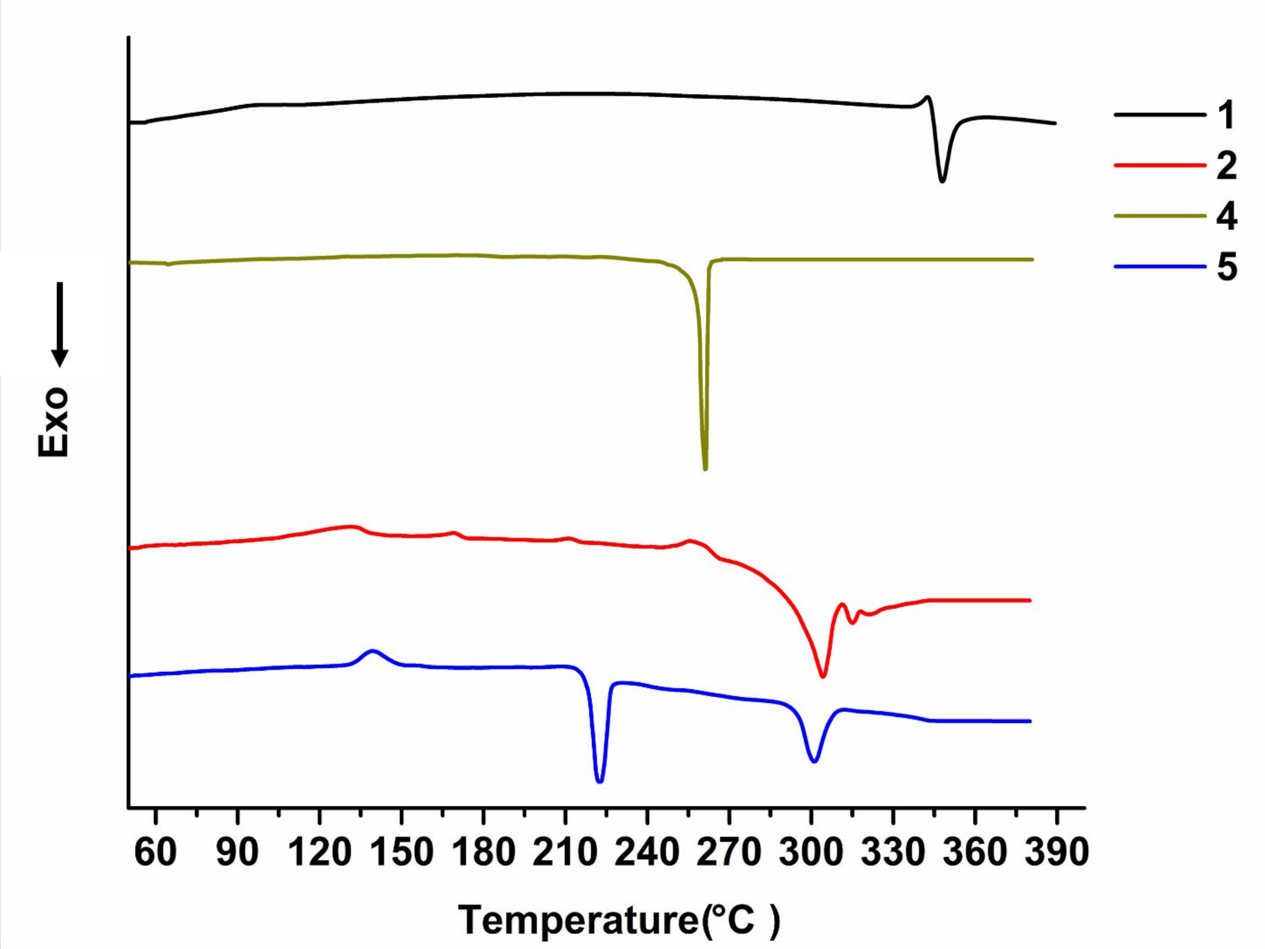

Fig. S6. DSC curves (N_2 atmosphere, heating rate 10 K/min), on heating, of compounds **1**, **2**, **4** and **5**.

From the TGA analysis reported in the typescript, decomposition temperatures (onset) of 347 °C, 230 °C, 270 °C, 210 °C were determined for **1**, **2**, **4** and **5** respectively. The DSC curves reported in Fig. S10 basically confirm the decomposition temperatures.

Computational Part (Energetics)

In order to be able to calculate the detonation parameters of **1** and of the corresponding salts, the enthalpy (H) was calculated quantum chemically with the CBS-4M method.[5] The CBS method begins with a HF/3–21G(d) calculation to optimize the structure and for the calculations of the zero-point energy. Then, using a larger basis set, the so-called base-energy is calculated. A MP2 /6 – 31 + G calculation with a CBS extrapolation gives the perturbation-theory correct energy, which takes the electron correlation into account. A MP4 (SDQ) /6 – 31 + (d,p) calculation is used, to estimate the correlation contributions of a higher order. The most widely used CBS-4M version today is a re-parametrization of the original CBS-4 version, which contains additional empirical correction terms (M stands for "minimal population localization" here).

The enthalpies of the gaseous species M can then be calculated using the method of the atomization energies:[6-8]

$$\Delta H_f^0(g,M) = H_M^0 - \sum H_{atoms}^0 + \sum \Delta H_f^0(atoms)$$

The calculated enthalpies (H) for neutral **1** and the corresponding ions as well as the relevant atoms H, C, N, O and Cl using the CBS-4M method, are summarized in Table S3.

Tab. S3. CBS-4M values

	Symmetry	NIMAG	$-H^{298}$ / a. u.
1H-$C_4H_7N_9$	C_s	0	648.458350
$[C_4H_8N_9]^+$	C_s	0	648.839050
$[C_4H_9N_9]^{2+}$	C_s	0	649.075205
$[C_4H_6N_9]^-$	C_1	0	647.936052
$N(NO_2)_2^-$	C_2	0	464.499541
ClO_4^-	T_d	0	760.171204
NO_3^-	D_{3h}	0	280.080429
K^+			599.035967
H			0.500991
C			37.786156
N			54.522462
O			74.991202
Cl			459.674555
K			599.187712

Therefore, in Table S3 we already have the $H°_{\text{(molecules)}}$ and $H°_{\text{(atom)}}$ values (given in a. u. = atomic units; 1 a. u. = 1 H = 627.089 kcal mol^{-1}). The values for $\Delta H_f^0(atoms)$ are easily obtained from the literature and are summarized in Table S4.

Tab. S4. Literature values for $\Delta H_f^0(atoms)$ (in kcal mol^{-1}).

	NIST[9]
H	52.1
C	171.3
N	113.0
O	59.6
Cl	29.0
K	21.3

According to the equation given above, we can now easily calculate the standard enthalpies of formation $\Delta H_f^0(g)$ for the gas phase (Tab. S5).

Tab. S5. Calculated standard enthalpies of formation $\Delta H_f^0(g)$ for the gas-phase.

gas-phase species	formula	$\Delta H_f^0(g)$/ kcal mol^{-1}	$\Delta H_f^0(g)$ / kJ mol^{-1}
**1**	$C_4H_7N_9$	120.0	502.2
**1**-cation	$[C_4H_8N_9]^+$	247.6	1035.8
**1**-dication	$[C_4H_9N_9]^{2+}$	465.7	1948.6
**1**-anion	$[C_4H_6N_9]^-$	81.3	340.1
Dinitramide anion	$[N(NO_2)_2]^-$	-29.2	-122.2
Perchlorate anion	$[ClO_4]^-$	-66.1	-276.6
Nitrate anion	$[NO_3]^-$	-74.6	-312.3
K^+	K^+	116.4	487.1

To be able to convert the standard enthalpies of formation $\Delta H_f^0(g)$ for the gas-phase into values for the condensed phase, for covalent molecules (NG) we additionally require the enthalpy of sublimation $\Delta H_{sub.}$ (for solids) or the enthalpy of vaporization $\Delta H_{vap.}$ (for liquids). Both values can be estimated using the Trouton's rule, in which T_m is the melting point of the solid and T_b is the boiling point of the liquid:[10]

$$\Delta H_{sub.}\ [\text{J mol}^{-1}] = 188\ T_m[\text{K}]$$

$$\Delta H_{vap.}\ [\text{J mol}^{-1}] = 90\ T_b[\text{K}]$$

Neutral triamine (compound **1** of the typescript) is a solid and has a m.p. of 351 °C (= 624 K). Therefore, the enthalpy of sublimation is calculated to be $\Delta H_{sub.}$(**1**) = 117 kJ mol^{-1} (28.0 kcal mol^{-1}).

In the case of ionic solids of the type AB, AB_2 or A_2B, the lattice energy (ΔU_L) and lattice enthalpy (ΔH_L) can be calculated by using the Jenkin's method.[11-14] Only the molecular volumes of the ions are required. These can be most easily obtained from single crystal X-ray diffraction data:

$$\Delta U_L = |z_+||z_-|v\left[\frac{a}{\sqrt[3]{V_M}} + \beta\right]$$

Here $|z_+|$ and $|z_-|$ are the dimensionless charges of the cations and anions and v is the number of ions *per* 'molecule' (2 for [**1**-cation][N_3O_4] and [K][**1**-anion], 3 for [**1**-dication][ClO_4]$_2$ and [**1**-dication][NO_3]$_2$. V_M is the volume in nm^3 of a formula unit (e.g. for all anhydrous: V_M([**1**-cation][N_3O_4]) = 0.256 nm^3, V([**1**-dication][ClO_4]$_2$) = 0.324 nm^3, V_M([**1**-dication][NO_3]$_2$) = 0.280

nm^3, V_M([K][**1**-anion]) = 0.200 nm^3). The constants α and β are dependent on the composition of the salt and are summarized in Table S6.

Tab. S6. The values for the constants α and β for calculating the lattice energy by using the Jenkin's method.

**salt type**	**α / kJ mol^{-1}**	**β / kJ mol^{-1}**
AB	117.3	51.9
AB_2	133.5	60.9
A_2B	165.3	–29.8

The lattice energy ΔU_L can easily be converted into the corresponding lattice enthalpy ΔH_L:

$$\Delta H_L(A_pB_q) = \Delta U_L + \left[p\left(\frac{n_A}{2}-2\right) + q\left(\frac{n_B}{2}-2\right)\right] RT$$

n_A, n_B = 3 for monoatomic ions, 5 for linear polyatomic ions, 6 for non-linear, polyatomic ions.

Therefore, the following values for the lattice energies and enthalpies can be written for the anhydrous salts (Tab. S7):

Tab. S7. Lattice energies and enthalpies.

Salt	Salt name from Chart 3	ΔU_L / kJ mol^{-1}	ΔH_L / kJ mol^{-1}
[**1**-cation][N_3O_4]	**2**	473.3	478.3
[**1**-dication][ClO_4]$_2$	**4**	1531.6	1539.0
[**1**-dication][NO_3]$_2$	**5**	1589.8	1597.2
$[K]^+$[**1**-anion]	**7**	505.0	506.2

The final solid-state enthalpies of formation ΔH_f^0 for all compounds in this study are summarized in Tab. S8.

Tab. S8. Enthalpies of formation of triamine compounds.

compound	formula	ΔH_f^0/ kJ mol^{-1}	ρ / g cm^{-3}
**1**	$C_4H_7N_9$	+385.2	1.629
[**1**-cation][N_3O_4]	$C_4H_8N_{12}O_4$	+435.3	1.837
[**1**-dication][ClO_4]$_2$	$C_4H_9N_9O_8Cl_2$	-143.6	1.908
[**1**-dication][NO_3]$_2$	$C_4H_9N_{11}O_6$	-273.2	1.773
[K][**1**-anion]	$C_4H_6N_9K$	+321.0	1.704

The detonation and combustion parameters of the triamine compounds were computed using the EXPLO5 code (version V6.05.02)[15-16] (Tab. S9).

Tab. S9. Detonation parameters.

Compound	Formula	ρ / g cm^{-3}	V_{oD} / m s^{-1}	p_{C-J} /kbar	Q_{ex} /kJ kg^{-1}
**1**	$C_4H_7N_9$	1.629	7681	194	-2378
[**1**-cation][N_3O_4]	$C_4H_8N_{12}O_4$	1.837	8948	311	-4496
[**1**-dication][ClO_4]$_2$	$C_4H_9N_9O_8Cl_2$	1.908	7033	191	-1711
[**1**-dication][NO_3]$_2$	$C_4H_9N_{11}O_6$	1.773	8092	246	-3289
[K][**1**-anion]	$C_4H_6N_9K$	1.704	6788	146	-1245

The sensitivities are listed in Tab. S10.

Tab. S10. Sensitivities of triamine compounds (grain size 100 – 500 μm)

Compound	IS / J	FS / N	ESD / J
**1**	> 40	> 360	1
[**1**-cation][N_3O_4]	4	> 360	0.1
[**1**-dication][ClO_4]$_2$	4	192	0.1
[**1**-dication][NO_3]$_2$	> 25	> 360	0.6
[K][**1**-anion]	n.d.	n.d.	n.d.

n.d. = not determined

Optimized Structures of tautomers/conformers

In the following we report the optimized geometries (xyz format) for the conformers of all tautomers computed both in gas and water.

1H *cis* GAS

N	-0.262924	-2.703204	-1.435773
N	-0.448205	-2.389924	-0.114407
C	-0.114495	-1.109664	-0.088401
N	0.251206	-0.580325	-1.311119
C	0.149491	-1.616626	-2.129135
C	-0.101072	-0.307976	1.126432
N	-0.066650	1.084860	1.120800
C	0.018189	1.434791	2.438215
N	0.032333	0.360000	3.202922
N	-0.031836	-0.737213	2.364640
N	0.000509	2.746197	2.849071
N	0.479458	-1.629647	-3.467018
H	-0.384855	-3.656309	-1.742853
N	-0.111456	2.018462	0.070777
H	0.379614	3.401808	2.176440
H	0.331278	2.874214	3.796000
H	-0.086768	-2.211932	-4.069552
H	0.637924	-0.705554	-3.845818
H	0.408732	1.615202	-0.707437
H	-1.080471	2.112842	-0.233788

1H *cis* WATER

N	-0.317680	-2.679146	-1.450515
N	-0.505471	-2.355662	-0.132437
C	-0.099834	-1.094312	-0.101159
N	0.320416	-0.586695	-1.304254
C	0.167439	-1.617178	-2.135509
C	-0.069113	-0.301320	1.121791
N	-0.064529	1.085287	1.134352
C	0.032409	1.432295	2.452291
N	0.079695	0.347519	3.208589
N	0.028772	-0.746517	2.353844
N	-0.005712	2.735567	2.869421
N	0.518192	-1.638327	-3.455106
H	-0.551655	-3.602757	-1.787100
N	-0.150493	2.024228	0.093466
H	0.291346	3.407599	2.170791
H	0.390369	2.894525	3.786971
H	0.065489	-2.322838	-4.046107
H	0.609580	-0.726445	-3.883135
H	0.384713	1.656240	-0.691963
H	-1.123931	2.087938	-0.204234

1H *trans* GAS

N	0.189794	-1.328091	-2.335911
N	0.175443	-0.226172	-1.518321
C	0.028601	-0.778522	-0.314194
N	-0.056738	-2.144883	-0.311377
C	0.054536	-2.454375	-1.590386
C	-0.033070	0.022653	0.897544
N	-0.046230	1.417178	0.909555
C	-0.056841	1.751433	2.234782
N	-0.051383	0.667362	2.986401
N	-0.031075	-0.419791	2.132746
N	-0.143761	3.053500	2.659497
N	0.097629	-3.730337	-2.106995
H	0.371101	-1.221920	-3.322116
N	-0.039724	2.365689	-0.125921
H	0.213989	3.738217	2.005282
H	0.138030	3.188857	3.620674
H	-0.144472	-4.431674	-1.418438
H	-0.359978	-3.876866	-2.996960
H	0.642514	2.063471	-0.818338
H	-0.948367	2.344269	-0.587522

1H *trans* WATER

N	0.148741	-1.344581	-2.333491
N	0.133502	-0.237384	-1.529044
C	0.025359	-0.775880	-0.318792
N	-0.032021	-2.143393	-0.294963
C	0.048903	-2.468696	-1.583106
C	-0.021964	0.032109	0.891956
N	-0.041762	1.420795	0.917686
C	-0.062903	1.754243	2.243991
N	-0.058694	0.663161	2.991304
N	-0.028089	-0.422156	2.124507
N	-0.156156	3.050446	2.669387
N	0.100606	-3.739931	-2.081664
H	0.233011	-1.246273	-3.335749
N	-0.025780	2.377950	-0.106589
H	0.159036	3.738611	1.995562
H	0.170142	3.211905	3.613255
H	-0.236825	-4.451810	-1.447067
H	-0.194935	-3.878016	-3.039120
H	0.734449	2.152386	-0.744459
H	-0.894621	2.306514	-0.633604

2H *cis* GAS

N	0.009528	-2.878240	-1.511246
N	0.065217	-2.396133	-0.233630
C	0.021846	-1.051499	-0.235964
N	-0.050016	-0.602587	-1.492320
C	-0.051071	-1.757355	-2.225309
C	0.028497	-0.250989	0.966215
N	0.100766	1.133618	0.987294
C	0.017243	1.461774	2.312525
N	-0.091220	0.373121	3.054888
N	-0.092222	-0.704367	2.192849
N	0.120298	2.754821	2.751528
N	-0.054086	-1.748317	-3.604956
N	0.224364	2.072329	-0.050048
H	0.099890	-3.016992	0.562050
H	-0.212656	2.899175	3.694776
H	-0.159481	3.459237	2.080342
H	-0.335505	-2.621223	-4.029947
H	-0.488079	-0.934145	-4.016646
H	1.191416	2.065788	-0.374226
H	-0.344728	1.741983	-0.828176

2H *cis* WATER

N	0.018116	-2.886266	-1.507417
N	0.058298	-2.399794	-0.230318
C	0.016628	-1.055231	-0.241186
N	-0.041212	-0.612681	-1.500090
C	-0.037220	-1.767369	-2.230769
C	0.012546	-0.249073	0.961240
N	0.097416	1.133147	0.993134
C	0.026685	1.460425	2.319238
N	-0.089932	0.365099	3.057504
N	-0.105822	-0.709965	2.185945
N	0.145046	2.745460	2.761546
N	-0.022051	-1.761827	-3.609946
N	0.220670	2.085440	-0.027887
H	0.096760	-3.023273	0.564256
H	-0.232535	2.918591	3.683821
H	-0.077151	3.456077	2.074210
H	-0.333977	-2.624678	-4.037673
H	-0.453139	-0.944969	-4.023814
H	1.152247	2.011117	-0.433775
H	-0.451374	1.859769	-0.758019

2H *trans* GAS

N	-0.131296	-1.540815	-2.544013
N	-0.126473	-0.503342	-1.659812
C	-0.088111	-0.965922	-0.388437
N	-0.067454	-2.295813	-0.394952
C	-0.099045	-2.593234	-1.723726
C	-0.078805	-0.114188	0.790689
N	0.026695	1.272358	0.757381
C	0.011213	1.654853	2.078503
N	-0.084279	0.602476	2.854713
N	-0.143976	-0.514114	2.039446
N	-0.025313	2.989800	2.460075
N	-0.147542	-3.894713	-2.177056
N	-0.003859	2.091033	-0.381465
H	-0.164529	0.459007	-1.964386
H	-0.174345	3.062704	3.461227
H	0.788679	3.533230	2.188515
H	0.162372	-4.028671	-3.129220
H	0.214041	-4.573224	-1.521550
H	-0.750706	2.775736	-0.270065
H	0.882732	2.582841	-0.475867

2H *trans* WATER

N	-0.078957	-1.526891	-2.535085
N	-0.065484	-0.490961	-1.647221
C	-0.080717	-0.954659	-0.383276
N	-0.109494	-2.290948	-0.392360
C	-0.107635	-2.586794	-1.722816
C	-0.071978	-0.114197	0.802094
N	-0.005901	1.269393	0.784996
C	-0.016149	1.644131	2.105478
N	-0.080539	0.571937	2.876819
N	-0.118264	-0.535588	2.045144
N	-0.058215	2.948692	2.516698
N	-0.198043	-3.883557	-2.187588
N	0.027084	2.068937	-0.363694
H	-0.042920	0.480276	-1.936503
H	0.091624	3.063950	3.511597
H	0.459120	3.623176	1.965519
H	0.134801	-4.014298	-3.134634
H	0.171627	-4.575602	-1.548167
H	-0.755118	2.720724	-0.350232
H	0.905155	2.582279	-0.406767

4H *cis* GAS

N	0.484192	-0.739671	1.829573
N	0.435079	-0.419415	3.170420
C	0.224423	0.875040	3.262369
N	0.144694	1.430500	1.996689
C	0.318121	0.375493	1.148096
C	0.059172	1.644985	4.483981
N	0.144119	3.032491	4.546114
C	-0.035153	3.335222	5.876884
N	-0.207975	2.238188	6.570896
N	-0.153173	1.170923	5.690269
N	-0.141208	4.650015	6.321804
N	0.392597	0.507363	-0.230650
N	0.229518	3.926597	3.466102
H	0.088356	2.421625	1.799246
H	-0.469581	4.663009	7.282284
H	0.718487	5.187320	6.249186
H	0.524139	-0.398015	-0.668036
H	-0.382981	1.004808	-0.654020
H	-0.496051	4.633506	3.582936
H	1.136520	4.390538	3.478799

4H *cis* WATER

N	0.311778	-0.776634	1.844582
N	0.223156	-0.453950	3.188414
C	0.177157	0.858979	3.279605
N	0.241553	1.417843	2.022663
C	0.323804	0.361776	1.165971
C	0.062298	1.630403	4.505369
N	0.086046	3.015240	4.563814
C	-0.031862	3.319819	5.898570
N	-0.119007	2.209858	6.607247
N	-0.063270	1.143874	5.718918
N	-0.144559	4.604100	6.364922
N	0.484281	0.488394	-0.192321
N	0.163733	3.881174	3.465860
H	0.237773	2.416434	1.837709
H	-0.087761	4.668459	7.374240
H	0.423819	5.305370	5.904292
H	0.317567	-0.373326	-0.697544
H	0.025102	1.282082	-0.622181
H	-0.641027	4.505062	3.464222
H	1.022711	4.425566	3.508588

4H *trans* GAS

N	-1.285696	-0.204357	-2.591411
N	-0.194019	-0.125703	-1.742888
C	-0.651554	0.017851	-0.517241
N	-2.027154	0.047775	-0.526695
C	-2.365696	-0.092216	-1.845747
C	0.117436	0.104943	0.699423
N	1.501125	0.108425	0.755617
C	1.789903	0.152304	2.093976
N	0.680301	0.179735	2.809072
N	-0.376121	0.143353	1.915370
N	3.076578	0.235157	2.556765
N	-3.666453	-0.032953	-2.308636
N	2.468307	0.054081	-0.260655
H	-2.618386	0.205280	0.277941
H	3.780324	-0.139534	1.932896
H	3.183897	-0.013131	3.530392
H	-3.712797	-0.207485	-3.305609
H	-4.339263	-0.591485	-1.797054
H	2.154312	-0.628088	-0.949735
H	2.482628	0.955253	-0.737640

4H *trans* WATER

N	-1.280767	-0.129884	-2.618479
N	-0.189022	-0.065159	-1.766313
C	-0.648326	0.022879	-0.535955
N	-2.024451	0.024020	-0.547338
C	-2.368909	-0.072722	-1.864849
C	0.120147	0.091434	0.686014
N	1.502691	0.111856	0.758953
C	1.785329	0.158378	2.098215
N	0.666222	0.169267	2.804770
N	-0.385053	0.122604	1.898403
N	3.063341	0.257293	2.568984
N	-3.660721	-0.028317	-2.321280
N	2.488251	0.069207	-0.235516
H	-2.634408	0.098190	0.256353
H	3.778319	-0.066074	1.927922
H	3.191194	-0.043601	3.526212
H	-3.759768	-0.319415	-3.286020
H	-4.365096	-0.433211	-1.717196
H	2.291367	-0.716850	-0.851465
H	2.427334	0.919311	-0.793273

1H-1'H *cis* GAS

N	-0.101063	-2.721479	-1.620326
N	-0.195131	-2.438881	-0.293641
C	-0.026298	-1.129471	-0.290894
N	0.160912	-0.545177	-1.517225
C	0.112961	-1.587962	-2.344216
C	-0.003523	-0.352389	0.938663
N	-0.069943	1.049895	0.953850
C	0.028228	1.442097	2.248764
N	0.127042	0.310006	2.954877
N	0.123932	-0.811362	2.152361
N	-0.001084	2.711558	2.651156
N	0.302853	-1.553307	-3.687222
H	-0.172937	-3.681570	-1.929065
N	-0.215898	1.998724	-0.067571
H	0.221145	0.196426	3.955121
H	-0.015158	3.427924	1.934255
H	0.143356	2.974544	3.614225
H	-0.012769	-2.317470	-4.265379
H	0.319407	-0.643083	-4.122735
H	0.432200	1.751687	-0.815295
H	-1.158230	1.919290	-0.449703

1H-1'H *cis* WATER

N	-0.173830	-2.709646	-1.632916
N	-0.303416	-2.409919	-0.308849
C	-0.020815	-1.116798	-0.299782
N	0.271599	-0.564365	-1.515235
C	0.165862	-1.604429	-2.343800
C	0.012031	-0.333783	0.928314
N	-0.082529	1.058041	0.950681
C	0.038428	1.447407	2.246652
N	0.179267	0.313858	2.945055
N	0.183923	-0.798821	2.135654
N	-0.015062	2.705499	2.663546
N	0.429046	-1.591179	-3.678592
H	-0.343013	-3.651419	-1.959942
N	-0.289722	1.995091	-0.067942
H	0.301999	0.213004	3.943887
H	-0.046802	3.433033	1.959938
H	0.189188	2.952813	3.620803
H	0.036836	-2.329024	-4.247590
H	0.418928	-0.677920	-4.112541
H	0.307793	1.738983	-0.852330
H	-1.259711	1.929575	-0.375010

1H-1’H *trans* GAS

N	-0.006673	-1.281320	-2.532924
N	-0.040256	-0.216775	-1.684282
C	0.026246	-0.821232	-0.501534
N	0.103502	-2.176099	-0.524447
C	0.078084	-2.446291	-1.825614
C	0.021128	-0.051054	0.731856
N	-0.070163	1.350469	0.777330
C	-0.044787	1.710672	2.085656
N	0.056805	0.564012	2.767131
N	0.099187	-0.537928	1.938504
N	-0.112315	2.967934	2.516819
N	0.076590	-3.685037	-2.375435
H	-0.074752	-1.131761	-3.530009
N	-0.175097	2.324359	-0.223068
H	0.103033	0.426548	3.767686
H	-0.186261	3.700188	1.820223
H	-0.091352	3.207316	3.496307
H	0.353317	-3.818677	-3.336313
H	0.268368	-4.450564	-1.744621
H	0.625177	2.233355	-0.847698
H	-1.009781	2.131886	-0.775567

1H-1’H *trans* WATER

N	-0.038819	-1.308465	-2.540725
N	-0.071808	-0.231024	-1.708615
C	0.009445	-0.807077	-0.516021
N	0.094369	-2.168470	-0.521713
C	0.060900	-2.457844	-1.821830
C	0.009118	-0.028963	0.713285
N	-0.066648	1.365097	0.770715
C	-0.042131	1.713323	2.085323
N	0.045140	0.559039	2.756632
N	0.077430	-0.530481	1.916570
N	-0.091943	2.959160	2.532020
N	0.053290	-3.708414	-2.358628
H	-0.093330	-1.179839	-3.542312
N	-0.155492	2.343523	-0.222787
H	0.087272	0.424693	3.758129
H	-0.172727	3.704216	1.851426
H	-0.096534	3.176027	3.517562
H	0.351970	-3.817441	-3.318436
H	0.368604	-4.442534	-1.738721
H	0.658952	2.269133	-0.829167
H	-0.987057	2.166343	-0.782707

2H-1'H *cis* GAS

N	-0.333313	-2.932961	-1.589852
N	-0.200382	-2.462415	-0.329703
C	-0.090761	-1.120195	-0.345778
N	-0.152222	-0.665412	-1.597849
C	-0.303825	-1.812725	-2.327436
C	0.080760	-0.292950	0.824488
N	0.087227	1.107384	0.779774
C	0.282203	1.545128	2.051230
N	0.375269	0.438713	2.800272
N	0.259189	-0.711556	2.048597
N	0.347895	2.826742	2.396388
N	-0.467011	-1.815550	-3.678114
N	-0.070276	2.018100	-0.271966
H	0.526544	0.362954	3.797301
H	-0.187190	-3.102189	0.454206
H	0.266548	3.516461	1.657605
H	0.508191	3.126914	3.346222
H	-0.159830	-1.001169	-4.187300
H	-0.377677	-2.701977	-4.153024
H	0.606255	1.787366	-0.998810
H	-0.997594	1.889336	-0.676253

2H-1'H *cis* WATER

N	-0.281741	-2.951341	-1.582128
N	-0.120038	-2.449455	-0.328738
C	-0.095315	-1.105992	-0.370141
N	-0.240938	-0.679169	-1.623855
C	-0.351526	-1.843567	-2.328322
C	0.070603	-0.266023	0.799113
N	0.094607	1.127488	0.769155
C	0.267265	1.546890	2.052545
N	0.338936	0.428327	2.787202
N	0.218358	-0.703492	2.019791
N	0.340177	2.814349	2.422992
N	-0.584243	-1.864664	-3.682525
N	-0.021122	2.048391	-0.274773
H	0.465062	0.349851	3.788081
H	-0.031513	-3.065672	0.468818
H	0.261898	3.525233	1.706203
H	0.468254	3.083367	3.387217
H	-0.256562	-1.045890	-4.178327
H	-0.345416	-2.732158	-4.145561
H	0.720682	1.874038	-0.949746
H	-0.917428	1.909490	-0.737006

2H-1'H *trans* WATER

N	0.188610	-2.292776	-2.111761
N	0.557995	-1.328498	-1.227319
C	-0.488793	-0.984418	-0.450342
N	-1.562632	-1.693159	-0.794684
C	-1.100820	-2.478617	-1.808807
C	-0.482571	-0.002630	0.626454
N	0.330332	1.133742	0.713866
C	-0.035838	1.796462	1.847293
N	-1.015636	1.062134	2.387564
N	-1.300045	-0.054262	1.641647
N	0.513693	2.924585	2.264116
N	-1.885619	-3.422998	-2.425163
N	1.306530	1.664240	-0.121541
H	-1.517764	1.234046	3.249051
H	1.523328	-1.031018	-1.196568
H	0.198699	3.396205	3.099314
H	1.227142	3.349774	1.684743
H	-1.568211	-3.716017	-3.340189
H	-2.878827	-3.233932	-2.389156
H	2.153851	1.104512	-0.104967
H	0.949791	1.803332	-1.062519

4H-1'H *cis* GAS

N	-2.440882	-0.184444	-1.950797
N	-2.018580	-0.213140	-0.664637
C	-0.708305	-0.054385	-0.657201
N	-0.244948	0.082025	-1.958720
C	-1.369945	-0.011956	-2.726210
C	0.077357	-0.006197	0.547238
N	1.488331	0.030010	0.595178
C	1.846934	0.033448	1.912827
N	0.700025	0.000777	2.585785
N	-0.406816	-0.015425	1.763936
N	3.099203	0.091112	2.384023
N	-1.386411	-0.010850	-4.087173
N	2.334634	0.062602	-0.509042
H	0.719690	0.138088	-2.252928
H	0.559047	-0.000173	3.588048
H	3.283121	0.056469	3.376694
H	3.890941	-0.004769	1.764902
H	-2.315894	-0.011042	-4.488343
H	-0.712844	0.551373	-4.587777
H	2.898999	0.908785	-0.526695
H	2.892711	-0.784696	-0.584999

4H-1’H *cis* WATER

N	-2.465967	-0.090301	-1.960255
N	-2.067686	-0.110264	-0.646023
C	-0.752251	-0.048837	-0.626430
N	-0.263396	0.007022	-1.911947
C	-1.366233	-0.020512	-2.708533
C	0.060233	-0.031669	0.573581
N	1.455202	0.025751	0.582965
C	1.848453	0.023444	1.892009
N	0.712512	-0.034007	2.594582
N	-0.404754	-0.068043	1.793793
N	3.098237	0.070869	2.333379
N	-1.329647	-0.052548	-4.073528
N	2.261040	0.075485	-0.552210
H	0.719157	0.045646	-2.161903
H	0.607644	-0.052583	3.600953
H	3.302629	0.063830	3.322747
H	3.878117	0.107189	1.691822
H	-2.219010	0.141277	-4.516187
H	-0.549021	0.407675	-4.523886
H	2.796606	0.940386	-0.577800
H	2.864503	-0.742201	-0.603021

4H-1’H *trans* GAS

N	-1.269383	0.067072	-2.706293
N	-0.241901	0.074572	-1.818816
C	-0.745619	0.011017	-0.600944
N	-2.123298	-0.037661	-0.663377
C	-2.401701	0.004322	-2.002796
C	0.035716	-0.016449	0.600954
N	1.436929	0.040924	0.600681
C	1.836823	-0.005377	1.898022
N	0.712906	-0.085326	2.619597
N	-0.419576	-0.093434	1.825346
N	3.108746	0.027730	2.283878
N	-3.646670	0.044452	-2.535321
N	2.375961	0.129581	-0.433930
H	-2.770150	-0.042296	0.113955
H	0.611558	-0.137497	3.623885
H	3.384797	-0.008821	3.253337
H	3.815781	0.088973	1.559692
H	-3.689523	-0.021156	-3.543620
H	-4.428277	-0.351968	-2.034838
H	2.236406	-0.660335	-1.063994
H	2.180474	0.971676	-0.975415

4H-1'H *trans* WATER

N	-1.317993	0.104682	-2.751067
N	-0.245919	0.118421	-1.892580
C	-0.714044	0.042474	-0.664742
N	-2.088246	-0.018248	-0.684508
C	-2.419771	0.023233	-2.006634
C	0.070382	0.010706	0.545014
N	1.463802	0.054517	0.582577
C	1.826977	-0.005165	1.893129
N	0.680230	-0.078660	2.579573
N	-0.420835	-0.069971	1.753315
N	3.078412	0.019388	2.323628
N	-3.697267	0.054313	-2.478434
N	2.425437	0.137857	-0.426297
H	-2.710588	-0.067544	0.112332
H	0.558089	-0.128943	3.582392
H	3.309695	-0.076602	3.301211
H	3.817514	0.051682	1.632370
H	-3.783556	-0.125072	-3.470608
H	-4.413137	-0.390979	-1.919558
H	2.306918	-0.651862	-1.057811
H	2.273900	0.995773	-0.953299

1H-4H *cis* GAS

N	-2.740950	-0.163835	-1.492315
N	-2.483483	-0.159803	-0.139435
C	-1.181813	-0.060284	-0.065052
N	-0.616422	-0.009787	-1.341390
C	-1.628343	-0.077238	-2.233369
C	-0.391527	0.018694	1.140599
N	1.000221	0.008223	1.176104
C	1.303907	0.095049	2.515621
N	0.190608	0.138031	3.228319
N	-0.865499	0.093703	2.362025
N	2.581492	0.240429	2.986603
N	-1.529854	-0.069764	-3.568722
N	1.860082	0.077739	0.065836
H	0.390047	0.070486	-1.491265
H	-3.700177	-0.234789	-1.806193
H	2.610803	0.348244	3.994819
H	3.283287	-0.408253	2.649960
H	-2.352221	-0.104545	-4.153378
H	-0.638232	0.043847	-4.027328
H	2.453253	0.903683	0.159284
H	2.454823	-0.749829	0.039275

1H-4H *cis* WATER

N	-2.735604	-0.125601	-1.493566
N	-2.481527	-0.096569	-0.143167
C	-1.176902	-0.030974	-0.068199
N	-0.605775	-0.019543	-1.329220
C	-1.612632	-0.080983	-2.229298
C	-0.400489	0.029183	1.154350
N	0.983174	0.065745	1.197055
C	1.292332	0.109281	2.533542
N	0.176981	0.097184	3.252044
N	-0.884332	0.049437	2.373735
N	2.561334	0.242217	3.012508
N	-1.506714	-0.095713	-3.553837
N	1.828532	0.088774	0.080381
H	0.402075	0.031062	-1.483719
H	-3.688917	-0.179428	-1.826731
H	2.643722	0.102748	4.011834
H	3.312469	-0.186045	2.485955
H	-2.328184	-0.134574	-4.140097
H	-0.603048	-0.052385	-4.002092
H	2.405739	0.927612	0.100756
H	2.417768	-0.741426	0.067764

1H-4H *trans* GAS

N	-1.497076	-0.163844	-2.446868
N	-0.263910	-0.129989	-1.821999
C	-0.559511	-0.038266	-0.552213
N	-1.943248	-0.013084	-0.363801
C	-2.518403	-0.094202	-1.579697
C	0.273484	0.034524	0.617583
N	1.649930	0.049235	0.723212
C	1.877810	0.107050	2.080126
N	0.726708	0.116315	2.743694
N	-0.270131	0.071534	1.816092
N	3.115192	0.237279	2.628228
N	-3.827332	-0.103090	-1.853271
N	2.551584	0.116914	-0.336583
H	-2.378090	0.045799	0.552948
H	-1.534702	-0.229439	-3.455146
H	3.899431	-0.216032	2.179641
H	3.131656	0.207187	3.639859
H	-4.164712	-0.167159	-2.802551
H	-4.515410	-0.038752	-1.117215
H	3.141460	0.940803	-0.248741
H	3.105270	-0.732781	-0.403298

1H-4H *trans* WATER

N	-1.461952	-0.203404	-2.412550
N	-0.262482	-0.179328	-1.739555
C	-0.603798	-0.047852	-0.484959
N	-1.984175	0.009971	-0.349848
C	-2.516628	-0.089924	-1.588769
C	0.235617	0.024648	0.689685
N	1.613059	0.071896	0.747535
C	1.900070	0.111614	2.090553
N	0.778354	0.087608	2.799909
N	-0.265327	0.032708	1.904762
N	3.163534	0.255618	2.580052
N	-3.802955	-0.067361	-1.917791
N	2.486059	0.151768	-0.338473
H	-2.483027	0.107190	0.527365
H	-1.473585	-0.302086	-3.419036
H	3.918160	-0.171368	2.057196
H	3.240759	0.117898	3.579900
H	-4.093011	-0.175626	-2.879257
H	-4.521253	-0.007744	-1.210812
H	3.084809	0.967858	-0.234872
H	3.047770	-0.694082	-0.401036

References

[1] Biedermann G.; Sillén, L. G. *Ark. Kemi.* **1953**, *40*, 425-440.

[2] Gran, G. *Analyst* **1952**, *77*, 661–671.

[3] Rossotti, F. J. C.; Rossotti, H. S. *The Determination of Stability Constants and Other Equilibrium Constants in Solution*, McGraw-Hill, New York, 1961.

[4] Gans, P.; Sabatini, A.; Vacca, A. *Talanta* **1996**, *43*, 1739-1753.

[5] Gaussian 16, Revision C.01, M. J. Frisch, G. W. Trucks, H. B. Schlegel, G. E. Scuseria, M. A. Robb, J. R. Cheeseman, G. Scalmani, V. Barone, G. A. Petersson, H. Nakatsuji, X. Li, M. Caricato, A. V. Marenich, J. Bloino, B. G. Janesko, R. Gomperts, B. Mennucci, H. P. Hratchian, J. V. Ortiz, A. F. Izmaylov, J. L. Sonnenberg, D. Williams-Young, F. Ding, F. Lipparini, F. Egidi, J. Goings, B. Peng, A. Petrone, T. Henderson, D. Ranasinghe, V. G. Zakrzewski, J. Gao, N. Rega, G. Zheng, W. Liang, M. Hada, M. Ehara, K. Toyota, R. Fukuda, J. Hasegawa, M. Ishida, T. Nakajima, Y. Honda, O. Kitao, H. Nakai, T. Vreven, K. Throssell, J. A. Montgomery, Jr., J. E. Peralta, F. Ogliaro, M. J. Bearpark, J. J. Heyd, E. N. Brothers, K. N. Kudin, V. N. Staroverov, T. A. Keith, R. Kobayashi, J. Normand, K. Raghavachari, A. P. Rendell, J. C. Burant, S. S. Iyengar, J. Tomasi, M. Cossi, J. M. Millam, M. Klene, C. Adamo, R. Cammi, J. W. Ochterski, R. L. Martin, K. Morokuma, O. Farkas, J. B. Foresman, and D. J. Fox, Gaussian, Inc., Wallingford CT, 2016.

[6] Curtiss, L. A.; Raghavachari, K.; Redfern, P. C.; Pople, J. A. *J. Chem. Phys*. **1997**, *106*, 1063.

[7] Byrd, E. F. C.; Rice, B. M. *J. Phys. Chem. A.* **2006**, *110*, 1005.

[8] Rice, B. M.; Pai, S. V.; Hare, J. *Combustion and Flame* **1999**, *118*, 445.

[9] Linstrom, P. J.; Mallard, W. G. (eds.), NIST Chemistry WebBook, NIST Standard Reference Database Number 69, June **2005,** National Institute of Standards and Technology, Gaithersburg MD, 20899 (http://webbook.nist.gov).

[10]Westwell, M. S.; Searle, M. S.; Wales, D. J.; Williams, D. H. *J. Am. Chem. Soc*. **1995**, *117*, 5013.

[11]Jenkins, H. D. B.; Roobottom, H. K.; Passmore, J.; Glasser, L. *Inorg. Chem*. **1999**, *38(16)*, 3609– 3620.

[12]Jenkins, H. D. B.; Tudela, D.; Glasser, L. *Inorg. Chem*. **2002**, *41(9)*, 2364– 2367.

[13]Jenkins, H. D. B.; Glasser, L. *Inorg. Chem.* **2002**, *41(17)*, 4378– 88.

[14]Jenkins, H. D. B. *Chemical Thermodynamics at a Glance*, Blackwell, Oxford, **2008**.

[15] Sućeska, M. EXPLO5 Version 6.05.02, Zagreb, **2018**.

[16] Klapötke, T. M. *Chemistry of High Energy Materials*, 5th edn., Walter de Gruyter, Berlin/Boston, **2019**, chapter 4.2.1, pp 143 – 146.
